# Cytotoxicity of *Gymnopilus purpureosquamulosus* extracts on hematologic malignant cells through activation of the SAPK/JNK signaling pathway

**DOI:** 10.1371/journal.pone.0252541

**Published:** 2021-05-28

**Authors:** Rich Milton Dulay, Benigno C. Valdez, Yang Li, Seemanti Chakrabarti, Braham Dhillon, Sofronio P. Kalaw, Renato G. Reyes, Esperanza C. Cabrera

**Affiliations:** 1 Center for Tropical Mushroom Research and Development, Department of Biological Sciences, College of Science, Central Luzon State University, Science City of Muñoz, Nueva Ecija, Philippines; 2 Department of Biology, College of Science, De La Salle University, Manila, Philippines; 3 Department of Stem Cell Transplantation and Cellular Therapy, UT MD Anderson Cancer Center, Houston, Texas, United States of America; 4 Department of Plant Pathology, University of Florida, Ft. Lauderdale Research and Education Center, Davie, Florida, United States of America; Chung Shan Medical University, TAIWAN

## Abstract

Treatment of hematologic malignancies is a formidable challenge for hematologists and there is an urgent need to identify safe and efficacious agents either via synthesis in the laboratory or isolation from natural products. Here, we report the cytotoxicity of extracts from mushroom *Gymnopilus purpureosquamulosus* Høil (*G*. *pps*) and describe its molecular mechanisms. Using leukemia, lymphoma and multiple myeloma cell lines, 28–35 ppm *G*. *pps* extract inhibited cell proliferation by ~46–79%, which correlates with activation of apoptosis as indicated by increase in annexin V-positive cells (~5–8-fold), production of reactive oxygen species (~2–3-fold), cells in sub G0/G1 phase (~3–13-fold), caspase 3 enzymatic activity (~1.6–2.9-fold), DNA fragmentation, PARP1 cleavage and down-regulation of prosurvival proteins. Mitochondrial membrane potential decreased and leakage of pro-apoptotic factors to cytoplasm was observed, consistent with the activation of intrinsic apoptosis. Western blot analysis showed activation of the ASK1-MEK-SAPK/JNK and ASK1-P38 MAPK pathways possibly due to changes in the cellular redox status as suggested by decreased protein levels of peroxiredoxin, thioredoxin and thioredoxin reductase. Moreover, antioxidant N-acetylcysteine alleviated the cytotoxicity of *G*. *pps*. Pharmacological inhibition of SAPK/JNK and P38 alleviated the *G*. *pps*-mediated cytotoxicity. The extract activated apoptosis in leukemia and lymphoma patient cell samples but not in mononuclear cells from healthy donors further supporting the therapeutic values of *G*. *pps* for hematologic malignancies.

## Introduction

Cancer is characterized by an uncontrolled proliferation of cells. It is the second leading cause of death globally, which accounts for approximately 18.1 million cases and 9.6 million deaths in 2018, and it is projected to rise by more than 60% over the next 20 years; breast and colorectal cancer are the most frequently diagnosed among cancer patients, and lung cancer is the leading cause of death (18.4%), followed by colorectal and stomach cancer [1]. Hematologic malignancies including leukemia, lymphoma and myeloma also afflict millions of children and adults, which are expected to account for 9.9% of the estimated 1.8 million new cancer cases diagnosed in the United States in 2020 [2]. Accordingly, cancer is considered as one of the major threats to humankind. Thus, there is an urgent need to address the increasing threat of this global burden.

Radiotherapy, chemotherapy, surgery, behavioral and dietary change, and the recent immunotherapy are the most common multidirectional approaches for cancers [3]. However, these approaches have significant adverse reactions during treatment such as pain, headaches, vomiting, diarrhea, malaise, fatigue, nausea, infections, mucositis, and rashes [4], and often lead to more serious and unwanted side effects including bone marrow suppression, neuro- and nephro-toxicities, gut epithelial cells sloughing, and hair loss [5–7]. For example, chemotherapeutic drug anthracyclines and cisplatin induce cardiotoxicity and nephrotoxicity, respectively [4]. These short- and long-term side effects concomitantly reduce the quality of life of the patients. Therefore, the search for effective and less toxic anticancer therapies has become an immense interest. Among such therapies is alternative and complementary medicine, which is essentially based on anticancer bioactive compounds and metabolites derived from natural sources such as plants, animals, algae, bacteria and mushrooms.

Medicinal mushrooms are considered potential natural sources of anticancer drugs. Some of the high-valued medicinal mushrooms with known anticancer properties include *Ganoderma lucidum*, *Agaricus blazei*, *Cordyceps militaris*, *Lentinula edodes*, *Schizophyllum commune*, *Grifola frondosa*, *Pleurotus ostreatus*, *Hericium erinaceus*, *Inonotus obliquus*, *Inocybe umbrinella*, *Coprinus comatus*, *Fomes fomentarius*, *Fomitopsis lilacinogilva*, *Gymnopilus junonius*, *Pycnoporus sanguineus*, *Trametes* spp., *Auricularia* spp., *Tremella* spp., and others [8–10]. Their anticancer properties are attributed to the presence of cytotoxic compounds like ganoderic acid, grifolin, ergosterol, trametenolic acid, inotodiol, lanostanes, ergosterol peroxide, hispidin, psilocin, psilocybin, bufotenin, aeruginascin, linolenic acid, eryngeolysin, ostreolysin, lecitins, laccases, beta-1,3-glucan, lentinan, and others [3]. In fact, there are several formulated pharmaceutical products derived from medicinal mushrooms. Polysaccharides such as schizophyllan, lentinan and krestin are some of these immunoceutical products, which are available in the world market [11].

Studies on the cytotoxic and/or anticancer activities of Philippine endemic and indigenous mushrooms are limited. Ergosterol peroxide and ergosterol from dichloromethane extracts of *Geastrum triplex* and *Termitomyces clypeatus*, respectively, exhibited cytotoxic activities against breast cancer (MCF-7), colon cancer (HT-29), and small lung cell carcinoma (H69PR) [12]. A water-insoluble polysaccharide extract from *Lenzites betulina* showed strong cytotoxic effects against K562 leukemia cells [13]. Moreover, ethanol extracts from commercially cultivated oyster mushroom (*Pleurotus ostreatus* var. *florida*) exhibited cytotoxicity against HT-29 colorectal cancer [14]. In our previous works on species listing and ethnomycological expedition, we reported that the Philippines has a rich macrofungal diversity [15–20], which remains pharmacologically unexplored.

In this regard, we evaluated the cytotoxicity of ethanol extracts of wild fruiting bodies of Philippine mushrooms against hematologic malignant cells. The molecular mechanisms of the cytotoxicity of *Gymnopilus purpureosquamulosus* extract were elucidated.

## Materials and methods

### Collection of mushroom materials

Wild mushrooms were collected within the vicinity of Central Luzon State University Campus, Science City of Muñoz, Nueva Ecija, Philippines during the first week of August 2020. Fruiting bodies were carefully picked from the substrate, placed in properly labeled paper bag, processed and morphologically identified. Molecular identification of two mushrooms, *G*. *purpureosquamulosus* and *Marasmiellus palmivorus*, using internal transcribed spacer (ITS), was initially done at the Molecular Biology Laboratory, Central Luzon State University.

### Molecular identification of *G*. *purpureosquamulosus*

The identity of *G*. *purpureosquamulosus* was confirmed independently at the University of Florida using the ITS region. Briefly, DNA was extracted using Qiagen DNeasy PowerSoil kit (Hilden, Germany). To extract DNA, 10 mg sample was homogenized using the FastPrep-24 bead beater (MP Biomedical, Irvine, CA, USA) for 1 min @ 5m/s. Steps were followed as per the DNA extraction protocol included with the kit and DNA was eluted in 20 μl autoclaved distilled water.

The ITS region was amplified using two fungus-specific primers, ITS1 and ITS4 [21] on the BioRad T100 Thermal Cycler (BioRad, Hercules, CA, USA). Polymerase chain reaction (PCR) was carried out in 30 μl reaction mixture containing 15 μl MangoMix (Meridian Bioscience, Cincinnati, OH, USA), 0.2 μM each primer (final concentration) and 50 ng DNA template. PCR cycling conditions consisted of an initial denaturation at 95°C for 3 min, followed by 35 cycles of 94°C for 30 sec, 54°C for 30 sec, and 72°C for 1 min and a final extension at 72°C for 10 min. The PCR products were electrophoresed on a 1.5% agarose gel stained with GelRed (Phenix Research Products, Candler, NC, USA) and visualized under UV light. Purification of the PCR products was done using the Zymo DNA Clean and Concentrator kit (Irvine, CA, USA) prior to sequencing. The nucleotide sequences were used to search the NCBI ‘nt’ and ‘ITS’ databases using BLASTN [22] to determine species identity.

### Preparation of mushroom ethanol extracts

Mushroom fruiting bodies were cleaned using a brush to remove dirt, cut into small pieces, air-dried at 25°C—30°C for 5–7 days and pulverized using a blender. Ten grams of each powdered mushroom was mixed with 100 mL 95% ethanol, tumbled at room temperature for 3 days, centrifuged at 1000 X *g* for 10 min and the resulting supernatant was filtered using Whatman #1 filter paper (GE Healthcare Life Sciences, Piscataway, NJ, USA). Each filtrate was vacuum-dried using Eppendorf Vacufuge Plus Concentrator System (Eppendorf, Enfield, CT, USA). The dried extracts were tared to constant weight and dissolved in dimethyl sulfoxide (DMSO) to prepare 50,000 ppm (parts per million) stock solutions.

### Cell culture

Cell lines used in this study included acute myeloid leukemia (AML), lymphoma and multiple myeloma. KBM3/Bu250^6^ or KBU, is an alkylating-agent resistant human AML cell line developed in our laboratory [23]; OCI-AML3 and MOLM13 AML cell lines were obtained from the laboratory of Dr. Michael Andreeff (UT MD Anderson Cancer Center, Houston, TX, USA). The lymphoma cell lines J45.01, Toledo, U937 and the multiple myeloma cell lines RPMI 8226, MM.1R and MC/CAR were purchased from the American Type Culture Collection (ATCC, Manassas, VA, USA). Cells were cultured in RPMI 1640 medium (Mediatech, Manassas, VA, USA) supplemented with 10% heat-inactivated fetal bovine serum (GeminiBio, Sacramento, CA, USA) and 100 U/mL penicillin and 100 μg/mL streptomycin (Mediatech) at 37°C in a fully humidified atmosphere of 5% CO_2_ in air. Absence of mycoplasma contamination was confirmed using the EZ-PCR mycoplasma detection kit (Biological Industries, Cromwell, CT, USA).

### Patient and healthy donor samples

Peripheral blood samples from patients were collected after obtaining written informed consent using UTMDACC Protocol Number PA12-0273 (A study to collect peripheral blood and bone marrow samples with hematological malignancies for pharmacological studies). Mononuclear cells were purified using lymphocyte separation medium (Mediatech), resuspended in freezing medium (50% heat-inactivated fetal bovine serum, 40% RPMI, 10% DMSO), and cryopreserved in liquid nitrogen until used. Mononuclear cells from healthy donors were purchased from ALLCELLS LLC (Almeda, CA, USA). For extract exposure, frozen purified cells were thawed at 37°C, washed with culture medium, and incubated overnight in suspension in the same complete RPMI 1640 culture medium used for cell lines, prior to mushroom extract treatment. Patient cell samples were exposed to DMSO (Control) or the indicated concentrations of *G*. *purpureosquamulosus* extract for 48 h. All studies were performed according to a protocol approved by the Institutional Review Board of the University of Texas MD Anderson Cancer Center, in accordance with the Declaration of Helsinki.

### Determination of IC_50_

Cells (50,000 in 100 μL) were exposed to different concentrations of mushroom extracts in 96-well plates (in triplicate) for 48 h prior to MTT assay (described below). The inhibition of cell proliferation was determined relative to the control cells exposed to solvent alone. The IC_50_ values (the concentration of mushroom extract that inhibited 50% proliferation) was calculated using the GraphPad Prism 8.0.0 software (San Diego, CA, USA).

### Cell proliferation and apoptosis assays

Cell suspensions (6 mL of 0.5 x 10^6^ cells/mL) in T25 flasks were exposed to mushroom extracts or solvent alone for 48 h, aliquoted (100 μL) in triplicate into 96-well plates and analyzed immediately for inhibition of proliferation by the MTT (3-(4,5 dimethylthiazol-2-yl)-2,5-diphenyl tetrazolium bromide) assay. Briefly, 30 μL of 2 mg/mL MTT reagent (Sigma-Aldrich, St. Louis, MO, USA) in phosphate buffer saline solution (PBS) was added per well and incubated for 3–4 h at 37°C. The solid reaction product was dissolved by adding 100 μL of solubilization solution (0.1 N HCl in isopropanol containing 10% Triton X-100), mixing, and incubating at 37°C for at least 1 h. Absorbance at 570 nm was measured using a Victor X3 (Perkin Elmer Life and Analytical Sciences, Shelton, CT, USA) plate reader. The number of MTT-positive cells was determined relative to the solvent control cells. Since the MTT reagent reacts with N-acetylcysteine (NAC), CCK-8 (Cell Counting Kit-8) assay (APExBIO, Houston, TX, USA) was performed in all experiments using NAC. Briefly, 100 μL cell culture was mixed with 10 μL CCK-8 reagent, incubated at 37°C for 2 h and absorbance was read using a plate reader (480 nm filter).

Like the MTT assay, apoptosis was determined immediately after the 48-h extract exposure by flow-cytometric measurements of phosphatidylserine externalization with Annexin-V-FLUOS (Roche Diagnostics, Indianapolis, IN, USA) and a fluorescent DNA-binding marker 7-aminoactinomycin D (BD Biosciences, San Jose, CA, USA) using a Muse Cell Analyzer (EMD Millipore, Billerica, MA, USA).

### Flow cytometric analysis of sub-G0/G1 DNA content

Cells in logarithmic growth phase (5 X 10^5^ cells/mL) were continuously incubated with the indicated concentrations of the extracts at 37°C for 48 h. Cells were centrifuged, resuspended in 70% ethanol in PBS, and fixed at -20°C overnight. Fixed cells were pelleted at 3000 X g at room temperature, washed with PBS, and treated with 0.25 mL 500 U/mL RNAse A in PBS containing 1.12% sodium citrate at 37°C for 30 min. After addition of 0.25 mL propidium iodide (50 μg/mL) solution, the cells were kept in subdued light for at least 1 h. The DNA content of at least 10,000 cells was analyzed using a Gallios Flow Cytometer (Beckman Coulter, Inc., Brea, CA, USA).

### Protein analysis

Western blot analysis was performed to determine extract-induced changes in the level of key proteins and their modifications. Cells were incubated with the extracts for 48 h, collected by centrifugation, washed with ice-cold PBS and lysed with lysis buffer (Cell Signaling Technology, Danvers, MA, USA). Total protein concentration was determined using the BCA protein Assay kit (Thermo Scientific, Rockford, IL, USA). The protein extracts were combined with the loading buffer containing dithiothreitol (Cell Signaling Technology), boiled for 5 min, and aliquots of equal amount of proteins were separated on polyacrylamide-SDS gels by electrophoresis. The proteins were transferred onto nitrocellulose membranes (Bio-Rad, Hercules, CA, USA). The relevant antibodies were added and detected using the chemiluminescent substrate Immobilon (EMD Millipore). The antibodies used, their dilutions and sources are presented in S1 Table.

### Preparation of cytoplasmic extracts

Since leakage of pro-apoptotic factors from mitochondria to cytoplasm is an early event, cells were exposed to solvent or *G*. *pps* extract for 24 h, collected and washed with ice-cold PBS. The cytoplasmic extracts were prepared using the Subcellular Protein Fractionation Kit for Cultured Cells (Thermo Scientific) and boiled with the loading buffer described above. Denatured protein samples were analyzed by Western blotting.

### Analysis of production of reactive oxygen species (ROS) and changes in mitochondrial membrane potential (MMP)

Cells exposed to mushroom extracts for 48 h were analyzed for the production of ROS using CM-H2DCFDA (5-(and-6)-chloromethyl-2′,7′-dichlorodihydrofluorescein diacetate, acetyl ester), an ROS indicator that diffuses into cells, where it is oxidized to a fluorescent product (Life Technologies, Grand Island, NY, USA). Briefly, cells were aliquoted (0.5 mL) into 5-mL tubes and 2 μL of 1.5 mM CM-H2DCFDA (freshly dissolved in DMSO) was added. Cells were incubated at 37°C for 1 h and immediately analyzed with a Gallios Flow Cytometer (Beckman Coulter, Inc.) using excitation/emission wavelengths of 492/520 nm. Geometric means of the fluorescence intensities were used in the analysis and the fold increase relative to the control was reported.

The JC-1 fluorescent probe (5,5′,6,6′-tetrachloro-1,1′,3,3′-tetraethylbenzimidazolyl-carbocyanine iodide) was used to determine changes in MMP using a JC-1 MMP assay kit (Cayman Chemical Co., Ann Arbor, MI, USA). Cells were exposed to mushroom extracts for 48 h and 0.5 mL cell suspension was aliquoted into 5 mL tubes. Diluted (1:10 with cell growth medium, 40 μL) MMP-sensitive JC-1 reagent was added to each tube, incubated at 37°C for 20 min, and immediately analyzed by flow cytometry (λex = 488 nm) using the 530-nm (FL-1 channel, green) and 585-nm (FL-2 channel, red) band-pass filters simultaneously. Healthy cells with functional mitochondria and high MMP exhibit red fluorescence (aggregated JC-1), whereas cells with disrupted mitochondria and low MMP show green fluorescence (monomeric JC-1).

### Caspase 3 assay

Cells were exposed to extracts for 48 h, harvested and washed with ice-cold PBS. Total cell extracts were prepared using the Caspase-3 Colorimetric Activity Assay kit (Chemicon International, Temecula, CA, USA). Total protein concentration was determined as described above. Equal amounts of protein were analyzed for caspase 3 activity using the same kit.

### Inhibition of SAPK/JNK and P38 signaling pathways

Cells were exposed to the indicated concentrations of SP600125, a broad spectrum JNK inhibitor, or PD169316, a P38 kinase inhibitor, (Selleck Chemicals LLC, Houston, TX, USA) for 1 h prior to addition of the *G*. *pps* extract. Cells were then analyzed after 48 h.

### Statistical analysis

Results are presented as the mean ± standard deviation of at least three independent experiments and statistical significance of the difference between two groups was determined using a Student’s paired t-test with a two-tailed distribution (Microsoft® Office Excel program). P values < 0.05 were considered statistically significant.

## Results

### *G*. *purpureosquamulosus* (*G*. *pps*) extract is cytotoxic towards hematologic malignant cells

**Fig 1:**
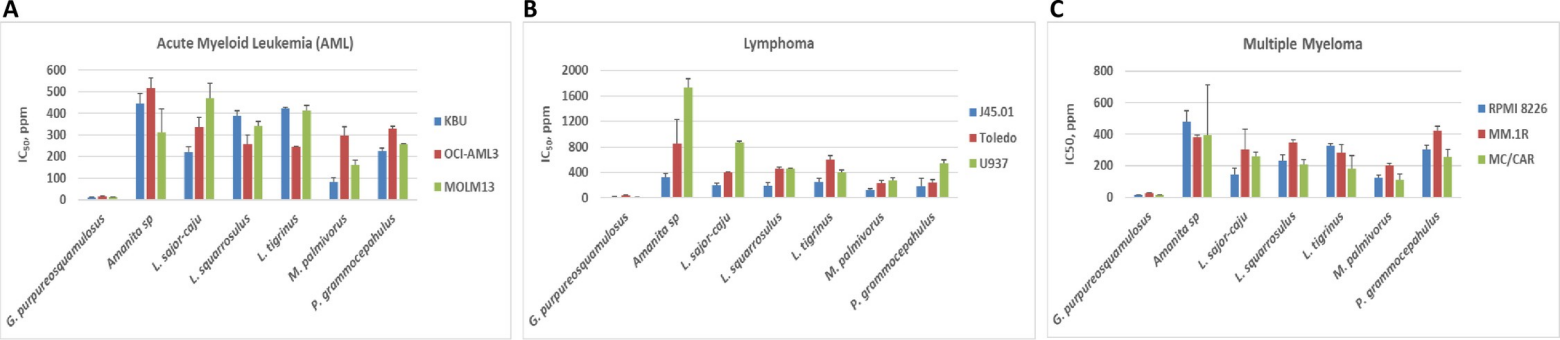
Comparison of the IC_50_ values of various mushroom extracts. Hematologic malignant cells (grouped by disease type) were exposed to different concentrations (ppm, parts per million) of mushroom ethanol extracts for 48 h and cell proliferation was determined by MTT assay. The concentrations that inhibited 50% cell proliferation (IC_50_) are shown. The results are average (± standard deviation) of three independent experiments; the MTT assay for each experiment was done in triplicate.

To compare the cytotoxicity of ethanol extracts of seven mushroom species, cells were exposed to various extract concentrations and their effects on cell proliferation were determined by calculating their IC_50_ values. Fig 1 shows the remarkable cytotoxicity of *G*. *pps* compared with extracts from *Amanita sp*, *Lentinus sajor-caju*, *Lentinus squarrosulus*, *Lentinus tigrinus*, *Marasmiellus palmivorus*, and *Polyporus grammocephalus*. The IC_50_ values of *G*. *pps* in the three AML cell lines (KBU, OCI-AML3 and MOLM13) range between 12–15 ppm while the IC_50_ values of the other six extracts in the same cell lines range between 81–516 ppm. *G*. *pps* extract is similarly cytotoxic in lymphoma (J45.01, Toledo, U937) and multiple myeloma (RPMI 8226, MM.1R, MC/CAR) cell lines with IC_50_ values of 13–41 ppm (Fig 1).

### *G*. *pps* extract inhibits cell proliferation and induces apoptosis

**Fig 2:**
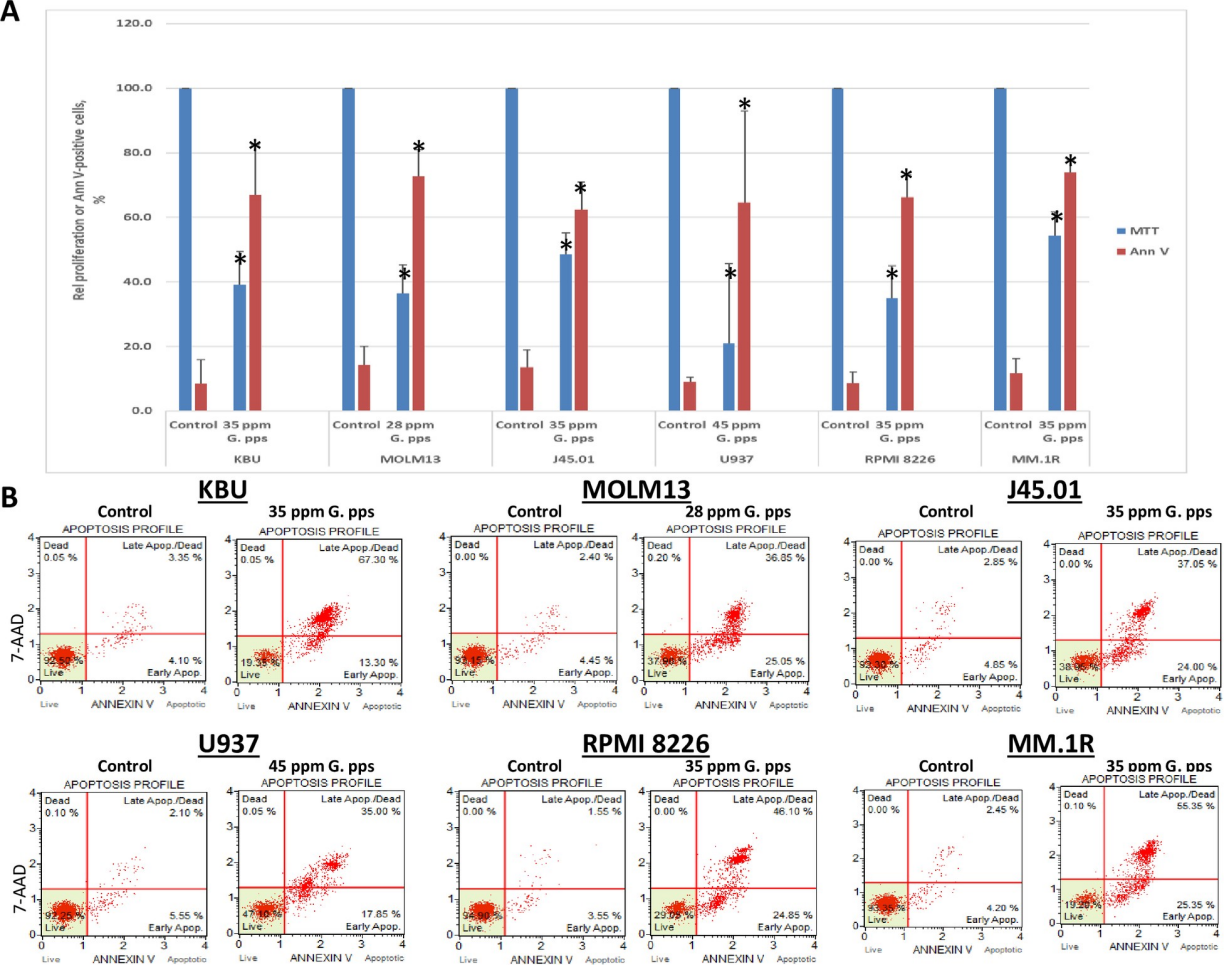
Extract from *Gymnopilus purpureosquamulosus* (*G*. *pps*) is cytotoxic towards hematologic malignant cells. (A) Leukemia, lymphoma and multiple myeloma cell lines were exposed to DMSO (Control) or the indicated concentrations of *G*. *pps* extract for 48 h and analyzed by MTT and annexin V (Ann V) assays. Rate of proliferation was calculated relative to control. The results are average (± standard deviation) of four independent experiments. For each experiment the MTT assay was done in triplicate and Ann V was done once. (B) Representative plots for Ann V assay by flow cytometry are shown (2,000 events). Asterisk (*) denotes *P*< 0.01 compared with control.

The sensitivity of leukemia, lymphoma and multiple myeloma cell lines to *G*. *pps* extract suggests its antitumor activity. To further determine its effects on cell survival and death, cells were exposed to extract concentrations greater than the calculated IC_50_ values for 48 h. MTT assay shows ~60% inhibition of cell proliferation in the presence of 28 ppm or 35 ppm *G*. *pps* in MOLM13 and KBU cell line, respectively, ~50% inhibition of J45.01 cells (35 ppm *G*. *pps*), ~80% inhibition of U937 (45 ppm G. *pps*), ~75% inhibition of RPMI 8226 (35 ppm *G*. *pps*) and ~45% inhibition of proliferation of MM.1R cells in the presence of 35 ppm *G*. *pps* (blue bars in Fig 2A).

The observed significant inhibition of cell proliferation mediated by the *G*. *pps* extract is consistent with the increase in Annexin-V positive cells, an indicator of programmed cell death or apoptosis. Exposure of the above-mentioned six cell lines to the indicated *G*. *pps* concentrations resulted in ~62–74% positivity compared with ~8–14% in the control cells (Fig 2A (red bars) and 2B).

### Sub G0/G1 population increases in cells exposed to *G*. *pps* extract

**Fig 3:**
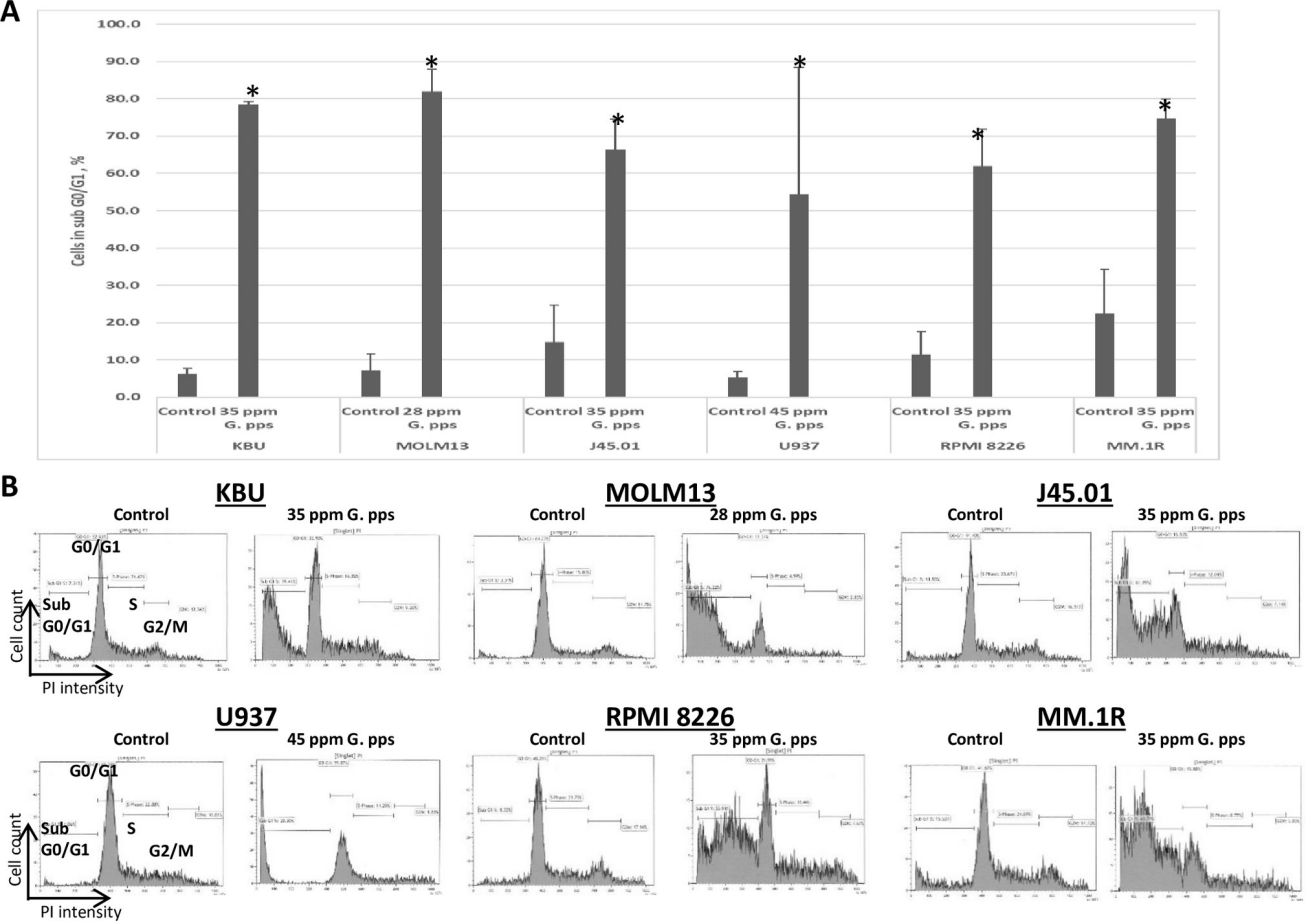
Extract from *Gymnopilus purpureosquamulosus* (*G*. *pps*) increases cells in sub G0/G1 phase. (A) Cells were exposed to DMSO (Control) or the indicated concentrations of *G*. *pps* extract for 48 h, fixed, washed, stained with propidium iodide and analyzed by flow cytometry. The results are average (± standard deviation) of three independent experiments. (B) Representative plots for cell cycle analysis by flow cytometry are shown (10,000 events). Asterisk (*) denotes *P* < 0.01 compared with control.

The *G*. *pps*-mediated increase in Annexin V-positive cells suggests increase in cell death. To further confirm this observation, cell cycle analysis was performed to determine changes in cell population in the sub G0/G1 phase, another indicator of cell death. Fig 3 shows a marked increase in sub G0/G1 phase from ~5–22% (control) to ~54–82% in cells treated with *G*. *pps* extract (Fig 3A and 3B).

### *G*. *pps* extract activates the apoptosis pathway

**Fig 4:**
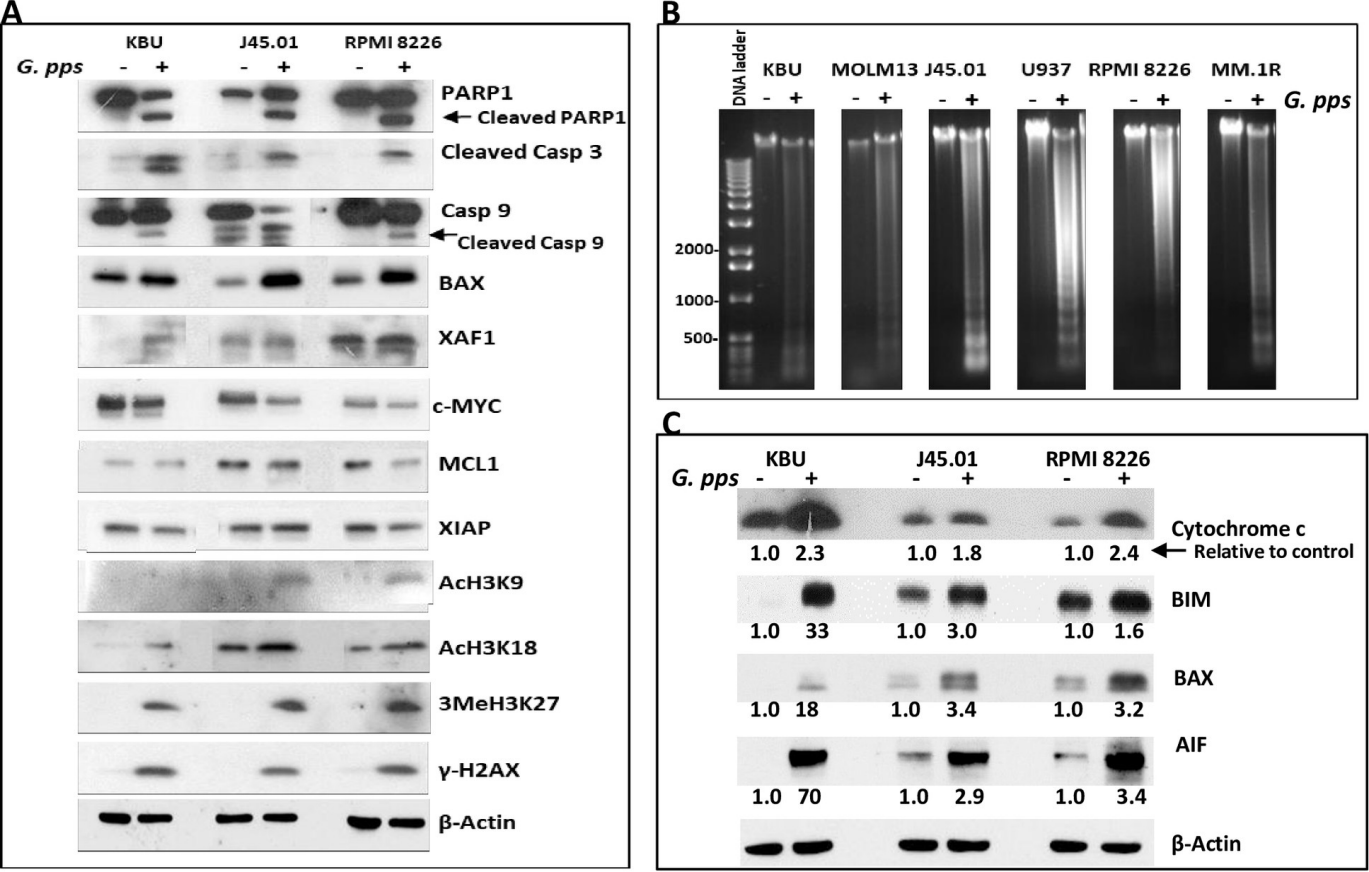
*Gymnopilus purpureosquamulosus* (*G*. *pps*) activates apoptosis. Cells were exposed to DMSO (-, Control) or 35 ppm *G*. *pps* extract (+) for 48 h and harvested. Proteins were analyzed by Western blotting (A) and genomic DNA by agarose gel electrophoresis (B). (C) Cytoplasmic extracts from cells exposed to solvent control (-) or *G*. *pps* (+) for 24 h were analyzed by Western blotting. Quantitative analysis of Western blot signals were done using the UN-SCAN-IT v 6.1 software. Signals were normalized relative to the β-Actin loading control and the calculated values were compared with untreated control cells (set to 1.0) as shown. The numbers indicate the fold difference between the control and treated cells.

The observed increase in Annexin V-positive cells and sub G0/G1 phase suggests activation of apoptosis. We, therefore, sought to confirm this by determining changes in the cleavage of PARP1 and caspase 3 which are commonly used molecular markers for apoptosis activation. Exposure of KBU, J45.01 and RPMI 8226 cells to *G*. *pps* extract resulted in extensive cleavage of PARP1 and caspase 3 (Fig 4A). This observation is further supported by the cleavage of caspase 9, another major player in the apoptosis pathway, and increased levels of pro-apoptosis proteins BAX and XAF1 with concomitant decrease in the levels of anti-apoptotic/prosurvival proteins c-MYC, MCL1 and XIAP1. The increased acetylation of histone 3 at Lys9 and 18 (AcH3K9 and AcH3K18), methylation of histone 3 at Lys27 (3MeH3K27) and phosphorylation of histone 2AX (γ-H2AX) all suggest chromatin relaxation and activation of the DNA-damage response mediated by *G*. *pps* extract (Fig 4A). Moreover, exposure of cells to *G*. *pps* extract increased DNA fragmentation ladder, a biochemical hallmark of apoptosis [24], as determined by agarose gel analysis (Fig 4B), suggesting activation of caspase-dependent DNase.

This activation of apoptosis might be due to damage of mitochondria. To determine if intrinsic apoptosis was involved, we compared the levels of mitochondrial proteins cytochrome c, BIM, BAX and apoptosis inducing factor (AIF) in the cytoplasmic extracts from cells exposed to solvent or *G*. *pps*; stressed mitochondria are known to release pro-apoptotic factors which trigger a cascade of events leading to activation of caspases. Fig 4C shows an increase in the level of cytochrome c (up to 2.4-fold), BIM (up to 33-fold), BAX (up to 18-fold), and AIF (up to 70-fold) in the cytoplasm of cells treated with *G*. *pps*, suggesting leakage of these apoptotic factors from mitochondria to cytoplasm.

**Fig 5:**
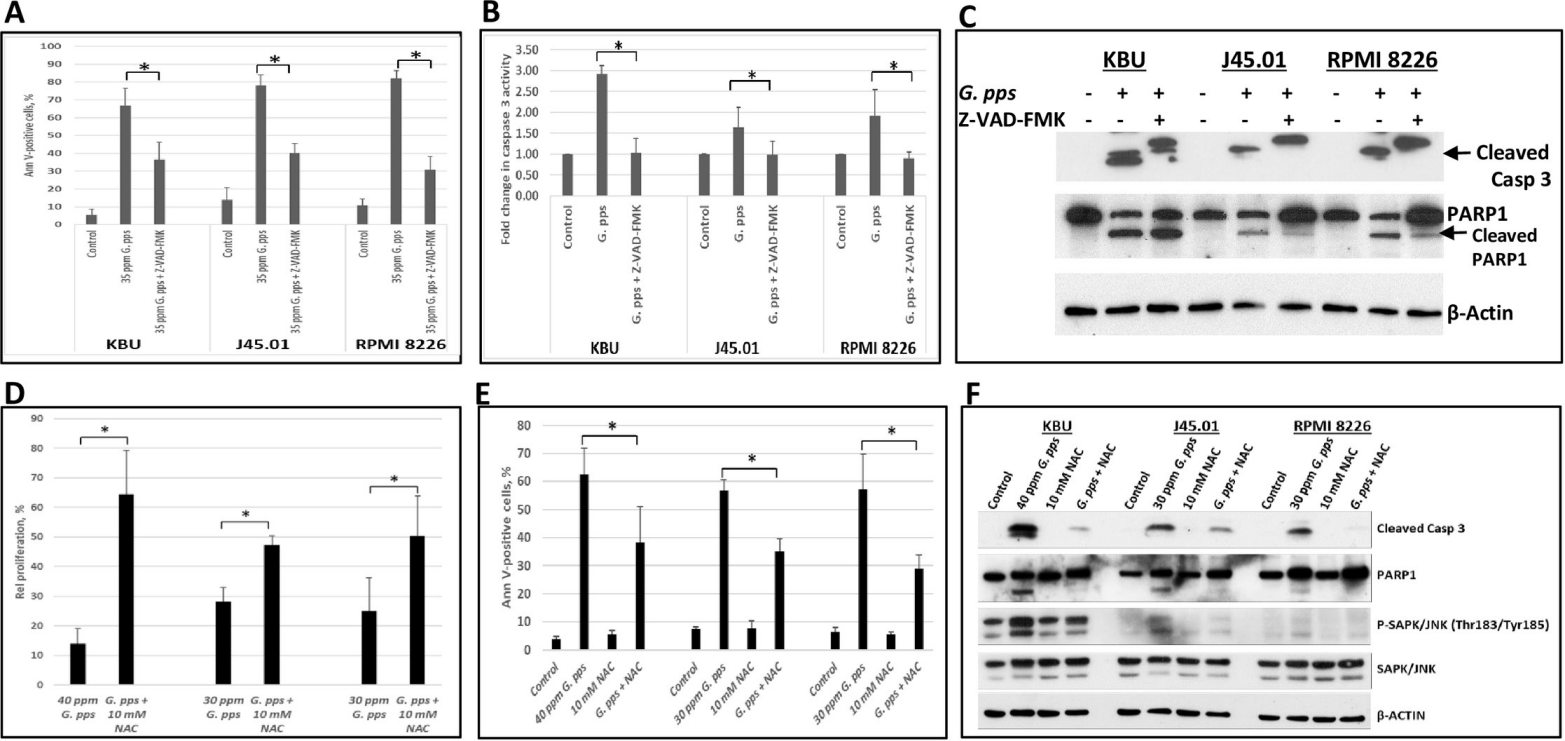
Pharmacological reversal of the cytotoxicity of *G*. *pps*. Cells were exposed to DMSO (-) or 35 ppm *G*. *pps* extract, without or with pan caspase inhibitor Z-VAD-FMK (40 μM), for 48 h and assayed for apoptosis (A) or caspase 3 enzymatic activity (B). The results (C and D) are average (± standard deviation) of three independent experiments. Cleavage of Caspase (Casp) 3 and PARP1 were determined by Western blotting (C). The relevance of reactive oxygen species was determined by exposing cells to *G*. *pps* extract in the absence or presence of N-acetylcysteine (NAC), and performing CCK-8 proliferation assay (D), Annexin V assay (E), and Western blot analysis (F). Cells were pre-exposed to NAC for 45 min prior to addition of *G*. *pps* extract. The results (D and E) are average of three independent experiments and F is a representative Western blot. Asterisk (*) denotes *P* < 0.01.

To further determine the role of caspases in the *G*. *pps*-mediated activation of apoptosis, cells were exposed to *G*. *pps* extract with or without Z-VAD-FMK (carbobenzoxy-valyl-alanyl-aspartyl-[O-methyl]- fluoromethylketone), a pan caspase inhibitor. Exposure of KBU cells to 35 ppm *G*. *pps* alone resulted in ~67% Annexin V-positive cells; addition of 40 μM Z-VAD-FMK significantly decreased Annexin V positivity to ~34% (Fig 5A). Similar alleviation in Annexin V positivity was obtained in J45.01 and RPMI 8226 cells exposed to *G*. *pps* in the presence of Z-VAD-FMK (Fig 5A). *In vitro* assay of the cell extracts revealed increased caspase 3 enzymatic activity in cells exposed to *G*. *pps* alone; exposure of cells to *G*. *pps* plus Z-VAD-FMK decreased caspase 3 activity closed to the control level (Fig 5B). These results are consistent with Western blot analysis. Increased activation of caspase 3 (by cleavage) was observed in cells exposed to *G*. *pps* alone; addition of Z-VAD-FMK inhibited this cleavage (Fig 5C). *G*. *pps*-mediated cleavage of PARP1 was also inhibited in cells exposed to *G*. *pps*+Z-VAD-FMK (Fig 5C). All these results underscore the relevance of caspases in the *G*. *pps*-mediated activation of apoptosis in leukemia, lymphoma and multiple myeloma cell lines.

### *G*. *pps* activates the production of reactive oxygen species (ROS) and decreases mitochondrial membrane potential (MMP)

To better understand the cellular responses underlying *G*. *pps*-mediated cell death, we examined the relevance of ROS, which are known cell-death mediators. ROS are known to be neutralized by antioxidants such as N-acetylcysteine (NAC). Fig 5D–5F show partial reversal of the *G*. *pps* cytotoxicity in KBU, J45.01 and RPMI 8226 cells when pre-exposed to NAC. The extract alone inhibited KBU cell proliferation by ~86%, J45.01 by ~72%, and RPMI 8226 by ~75%. Exposure to *G*. *pps*+NAC decreased these inhibitions to ~35%, ~53%, and ~50% in KBU, J45.01 and RPMI 8226 cells, respectively (Fig 5D). These results are consistent with Annexin V assay. Exposure of KBU, J45.01 and RPMI 8226 cells to *G*. *pps* alone resulted in ~62%, ~56%, and ~57% Annexin V positivity, respectively; pre-exposure to NAC significantly decreased these values to ~38%, ~35%, and ~29% (Fig 5E). Cleavage of caspase 3 and PARP1, and phosphorylation of SAPK/JNK were all alleviated when cells were pre-exposed to NAC prior to addition of *G*. *pps* extract (Fig 5F). These results indicate the involvement of ROS in the cytotoxicity of *G*. *pps*.

**Fig 6:**
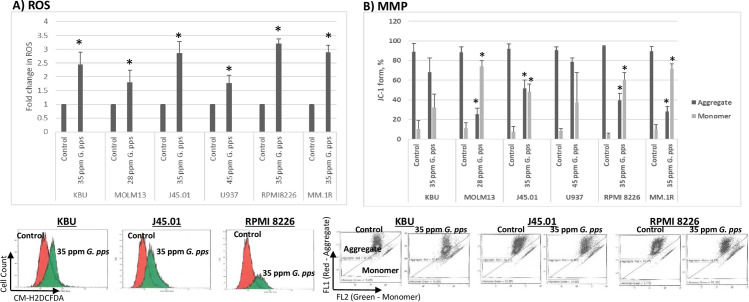
Extracts from *Gymnopilus purpureosquamulosus* (*G*. *pps*) increase the production of reactive oxygen species (ROS) and decrease the mitochondrial membrane potential (MMP). Cells were exposed to DMSO (Control) or the indicated concentrations of *G*. *pps* extract for 48 h, stained with either CM-H2DCFDA for ROS assay (A) or JC-1 reagent for MMP determination (B) by flow cytometry. The results are average (± standard deviation) of three independent experiments. Shown below the ROS bar graphs are representative plots (10,000 events) in the absence (red) or presence of *G*. *pps* extract (green), and below the MMP bar graphs are representative plots (10,000 events) for JC-1 aggregates and monomers. Asterisk (*) denotes *P*< 0.05 compared with control.

Next, we measured ROS production using the CM-H2DCFDA fluorescent probe. Exposure of KBU, MOLM13, J45.01, U937, RPMI 8226 and MM.1R cells to *G*. *pps* extract increased the production of ROS by ~1.75–3.2-fold relative to the control (Fig 6A). The results suggest that *G*. *pps* extract might have perturbed the mitochondrial metabolism resulting in enhanced generation of ROS.

To substantiate the effects of *G*. *pps* on the integrity of mitochondria, we analyzed changes in MMP, which plays a major role in programmed cell death. A JC-1 assay was used to determine changes in MMP. The aggregated form of JC-1 in the mitochondria emits a red fluorescence; a decrease in MMP causes translocation of the JC-1 probe to the cytoplasm, where it is converted into its monomeric form that emits a green fluorescence. As shown in Fig 6B, the control untreated cells showed ~88% - 95% aggregates and ~5% - 12% monomers, suggesting high MMP and good mitochondria integrity. In the presence of *G*. *pps*, the JC-1 aggregated form decreased to ~25%-79% and the monomeric form increased to ~32%-74%, suggesting a significant leakage of the JC-1 reagent from the mitochondria to the cytoplasm. Overall, these results suggest extensive depolarization of the mitochondrial membrane in cells exposed to the *G*. *pps* extract, which presumably caused the leakage of pro-apoptotic mitochondrial factors (Fig 4D) into the cytoplasm, thereby initiating the caspase-dependent cascade of events leading to apoptosis.

### Sensitivity of patient-derived leukemia and lymphoma cells to *G*. *pps* extract

**Fig 7:**
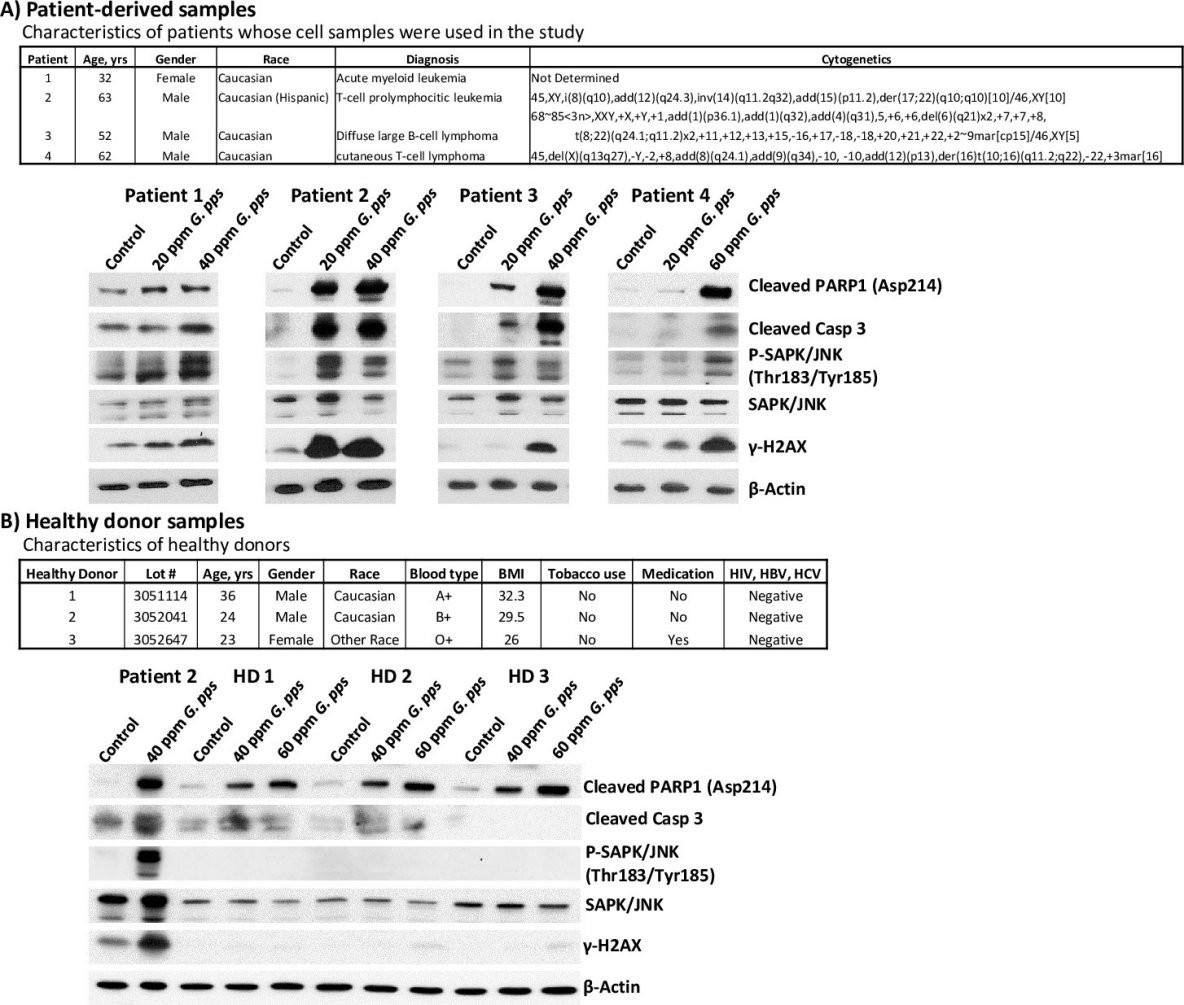
Cells derived from patients with hematologic diseases are sensitive to *Gymnopilus purpureosquamulosus* (*G*. *pps*) extract. Mononuclear cells isolated from peripheral blood samples of patients (A) and healthy donors (B) were exposed to DMSO (Control) or the indicated concentrations of *G*. *pps* extract for 48 h and analyzed by Western blotting. The characteristics of the patients (A) and healthy donors (B) are shown.

To assess the potential clinical implications of our observations, we isolated mononuclear cells (MNCs) from peripheral blood of patients with hematologic diseases and exposed the cells to the mushroom extract. Fig 7A (upper panel) shows the characteristics of the patients whose MNCs were used in this study. Exposure of the isolated MNCs to *G*. *pps* extract increased the cleavage of PARP1 and caspase 3 and phosphorylation of histone 2AX as shown by Western blot analysis (Fig 7A), consistent with what was observed in the cell lines (Fig 4). Mononuclear cells isolated from healthy individuals were less sensitive to *G*. *pps* extract; at concentration of 40 ppm *G*. *pps*, cleavages of PARP1 and caspase 3, and phosphorylations of SAPK/JNK and H2AX are significantly less in the mononuclear cells from three healthy donors compared with Patient 2 (Fig 7B). These observations suggest that the *G*. *pps* extract is highly cytotoxic to both leukemia and lymphoma cells but normal cells are more resistant.

### *G*. *pps* activates the ASK1-MEK-JNK signal transduction pathway

**Fig 8:**
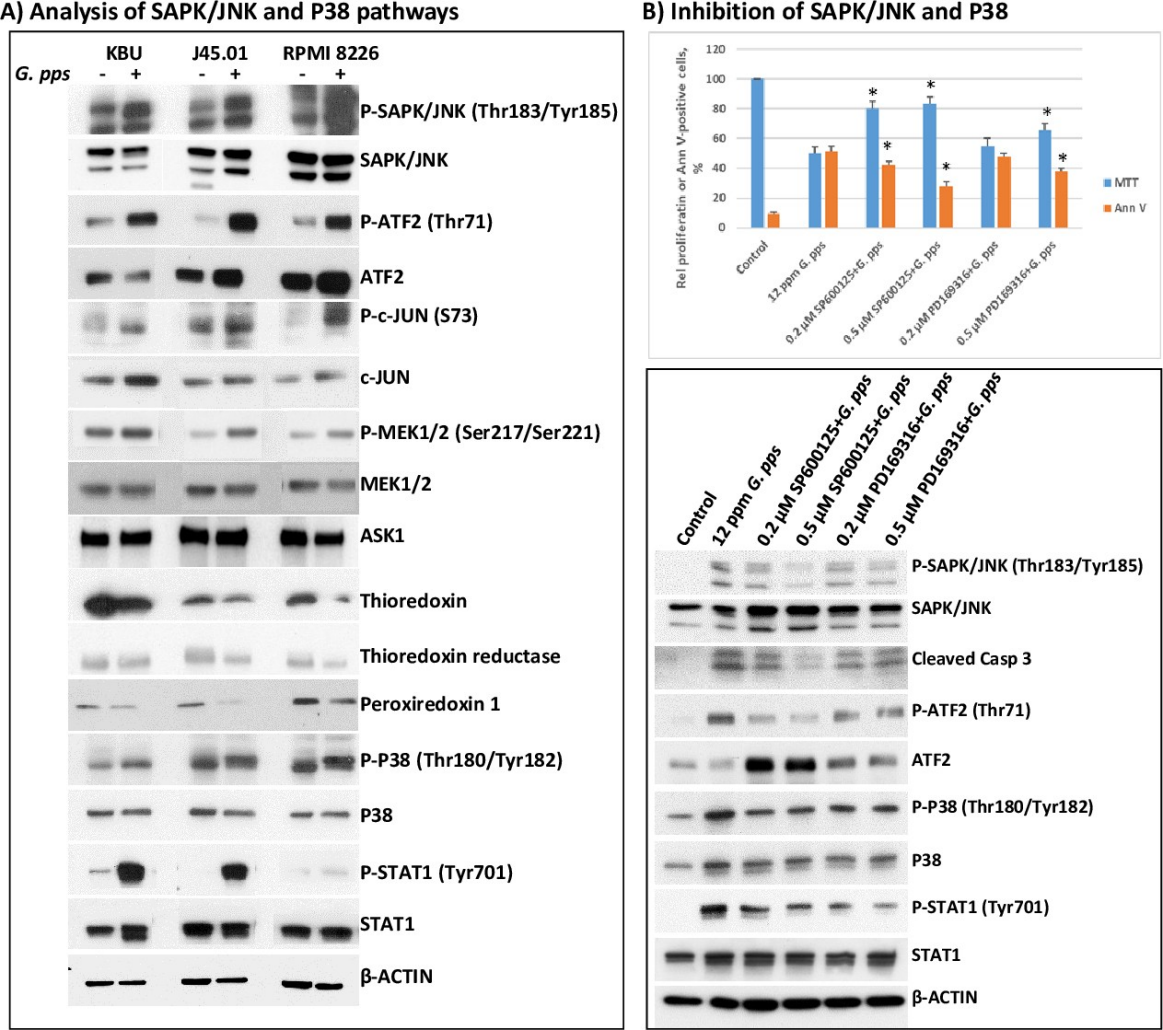
The stress-activated protein kinase/Jun-amino-terminal kinase (SAPK/JNK) is activated in cells exposed to extracts from *Gymnopilus purpureosquamulosus* (*G*. *pps*). (A) Leukemia (KBU), lymphoma (J45.01) and multiple myeloma (RPMI 8226) cell lines were exposed to DMSO (negative control) or 35 ppm *G*. *pps* extracts for 48 h and analyzed by Western blotting. (B) KBU cells were exposed to 12 ppm *G*. *pps* (IC_50_ value) alone or with 1 h-pre-exposure to SAPK/JNK inhibitor SP600125 or P38 inhibitor PD169316 and analyzed for proliferation (MTT), apoptosis (Ann V) and changes in the level of modifications of proteins involved in the SAPK/JNK and P38 signaling pathways. The MTT and Ann V results are average of three independent experiments and asterisk indicates statistically significant difference (*P* < 0.05) when compared with 12 ppm *G*. *pps* alone.

The observed increased production of ROS and decreased MMP (Fig 6) suggest a role for *G*. *pps*-mediated activation of stress pathways leading to apoptosis. We, therefore, sought to determine the effects of the mushroom extract on the activation of the stress-activated protein kinase/c-Jun N-terminal kinase (SAPK/JNK) signal transduction pathway, which is known to transmit and convert stress signaling into apoptosis signaling in various cell types [25]. Increased phosphorylation of SAPK/JNK at threonine 183/tyrosine185 was observed in KBU, J45.01 and RPMI 8226 cells exposed to *G*. *pps* extract (Fig 8A). This finding correlates with increased phosphorylation of its known substrates including activating transcription factor-2 (ATF2) and c-JUN.

Further analysis of proteins upstream of the SAPK/JNK pathway shows increased phosphorylation of MEK1/2 in cells treated with *G*. *pps* extract (Fig 8A), suggesting its involvement in the activation (by phosphorylation) of SAPK/JNK. Upstream of the MAP kinase family is the apoptosis signal-regulating kinase (ASK1) which is known to activate MEK by phosphorylation [26, 27]. The enzymatic activity of ASK1 is known to be inhibited by thioredoxin [28, 29]. Analysis of the level of ASK1 in *G*. *pps*-treated cells did not show significant changes relative to the control (Fig 8A). However, the level of thioredoxin protein decreased in *G*. *pps*-treated cells and it correlates with a decrease in the level of thioredoxin reductase protein (Fig 8A), which regulates the redox status of thioredoxin. Peroxiredoxin 1 is another antioxidant protein that mediates signal transduction; its level also decreased in cells treated with *G*. *pps* (Fig 8A). Taken together, our results suggest that the *G*. *pps* extract affects the cellular redox balance resulting in the activation of the ASK1-MEK-SAPK/JNK pathway in hematologic malignant cells.

To prove the relevance of the SAPK/JNK pathway, KBU cells were pre-exposed to 0.2 μM or 0.5 μM SP600125 for 1 hr prior to addition of 12 ppm *G*. *pps* extract, equivalent to its IC_50_ value in KBU cells. Analysis by MTT and Annexin V assays shows significant partial reversal of the effects of *G*. *pps* on the proliferation of KBU cells. Exposure of KBU cells to 12 ppm *G*. *pps* alone resulted in ~50% proliferation and ~51% Ann V-positive cells (Fig 8B); pre-exposure to 0.2 μM SP600125 increased proliferation to ~80% and decreased Ann V-positive cells to ~42%. At 0.5 μM SP600125, the proliferation and Ann V values were ~83% and ~28%, respectively. Pre-exposure to the SAPK/JNK inhibitor reduced caspase 3 cleavage and ATF2 phosphorylation, indicating inhibition of SAPK/JNK activity (Fig 8B, lower panel). The results suggest that inhibition of SAPK/JNK alleviates the cytotoxic effects mediated by the *G*. *pps* extract. The incomplete reversal of its cytotoxicity indicates possible involvement of other signaling pathways.

### *G*. *pps* activates the ASK1-P38 signaling pathway

ASK1 is also known to activate the P38 signaling pathway [30, 31]. We, therefore, determined if the *G*. *pps*-mediated activation of ASK1 upregulated the P38 pathway. Fig 8A shows increased phosphorylation of P38 at Thr180/Tyr182 in cells treated with *G*. *pps* extract. To further substantiate this observation, we examined the phosphorylation of STAT1, a known substrate for P38 and it mediates caspase-independent cell death [32]. Again, increased phosphorylation of STAT1 was observed in cells treated with *G*. *pps* extract (Fig 8A). As discussed above, phosphorylation of ATF2 was enhanced by *G*. *pps* extract; ATF2 is also a substrate for P38 [33]. These results indicate the activation of the ASK1-P38 pathway by *G*. *pps* extract in hematologic malignant cells.

Inhibition of the P38 kinase activity using 0.5 μM PD169316 significantly changed the proliferation rate and extent of apoptosis; the effects of 0.2 μM PD169316 were not significant (Fig 8B). Western blot analysis shows slight decrease in the phosphorylation of ATF2, less remarkable effects than SP600125. The level of P-STAT1 (Tyr701) decreased with 0.5 μM PD169316, which was probably due to P38 inhibition. The results suggest that the activation of the P38 pathway plays a minor contribution on the cytotoxicity mediated by *G*. *pps*.

## Discussion

**Fig 9:**
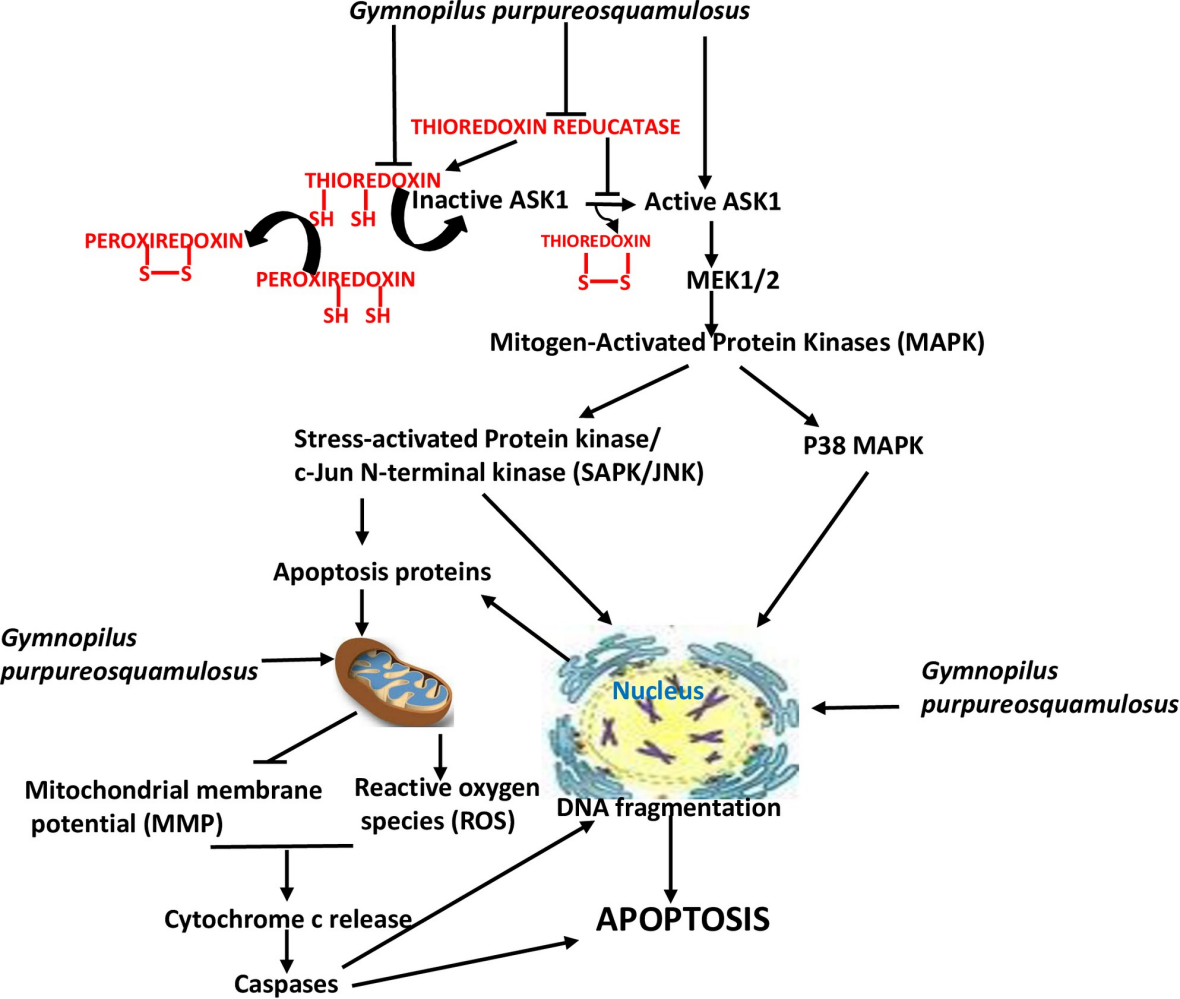
Suggested model for the cytotoxicity of *Gymnopilus purpureosquamulosus* extract on hematologic malignant cells.

The cytotoxicity of several mushroom extracts have been shown in various cancer cell models. However, the details of their mechanisms are less elucidated. Our present study is first to provide *in vitro* evidence on the cytotoxicity of ethanol extract of *Gymnopilus pupureosquamulosus* in leukemia, lymphoma and multiple myeloma cell lines and patient cell samples, and to propose a mechanism of its efficacy. The molecular mechanisms underlying the antineoplastic activity of the *G*. *pps* extract include perturbation of the cellular redox homeostasis resulting in the activation of the ASK1-MEK-SAPK/JNK stress signaling pathway, which eventually leads to the activation of intrinsic apoptosis. The observed apoptosis correlates with increased ROS production, mitochondrial membrane damage, activation of caspase 3, and DNA fragmentation. Prosurvival and antiapoptotic pathways are also compromised as substantiated by a decrease in the level of c-MYC, MCL1 and XIAP proteins. The complex interactions of the various pathways affected by the *G*. *pps* extract are illustrated in Fig 9.

Polyphenols, polysaccharides, proteins and their complexes present in mushroom extracts have been reported to be cytotoxic to various cancer cells [34–36]. Although the biologically active components in the *G*. *pps* extract remain to be identified, their cytotoxicity partly involves the activation of the ASK1 kinase, probably through changes in the cellular redox status. Exposure of cells to *G*. *pps* extract decreased the levels of thioredoxin, peroxiredoxin 1 and thioredoxin reductase (Fig 8A). Reduced thioredoxin is known to bind and inhibit ASK1 by promoting its ubiquitination and degradation [37]. Peroxiredoxin 1 is another antioxidant that regulates ASK1 activity [38]. On the other hand, thioredoxin reductase directly and indirectly inhibits ASK1 by keeping thioredoxin and peroxiredoxin 1 on their reduced form, and reducing the ASK1 disulfide linkages that bridge the active ASK1 multimer [39, 40]. The observed *G*. *pps*-mediated decrease in thioredoxin reductase (Fig 8A) must have compromised the stability and level of reduced thioredoxin and peroxiredoxin 1 with a concomitant upregulation of ASK1 kinase activity, consistent with increased phosphorylation of MEK1/2 (Fig 8A). This cascade of events leads to the activation of MAPK kinases, which phosphorylates SAPK/JNK and P38 (Figs 8A and 9), consistent with previous report [41]. The activated SAPK/JNK is known to translocate to the nucleus where it phosphorylates transcription factors including ATF2 and c-JUN (Fig 8A, illustrated in Fig 9), which may upregulate the expression of pro-apoptotic genes [42].

*BAX* is one of the c-JUN-target genes whose gene product forms oligomers and translocates from the cytosol to the mitochondrial membrane and induces its permeabilization resulting in the release of cytochrome c along with other pro-apoptotic proteins [43]. Indeed, exposure of leukemia, lymphoma and multiple myeloma cell lines to *G*. *pps* extract resulted in increased BAX expression and leakage of cytochrome c to the cytoplasm (Fig 4C), most likely due to decreased mitochondrial membrane potential (Fig 6B). BIM and AIF are two other pro-apoptotic mitochondrial proteins observed to leak into the cytoplasm in cells exposed to *G*. *pps* (Fig 4C).

The released cytochrome c is known to associate with procaspase 9/Apaf 1 to form the apoptosome complex leading to the cleavage and activation of caspase 9, which further triggers the activation of executor caspase 3 (by cleavage). The activated caspase 3 is known to translocate to the nucleus and it mediates caspase-dependent PARP1 cleavage and DNA fragmentation (Fig 4), both hallmarks of apoptosis [44]. These events are then communicated to the mitochondria through complex signaling pathways that decrease the levels of NAD+ and acetyl-CoA [45] and further amplify the activation of apoptosis. These mechanisms are all consistent with the observed *G*. *pps*-mediated death of tumor cells used in this study; cleavage of caspase 3 and PARP1 in cell lines and patient-derived cell samples increased, and DNA fragmentation occurred (Figs 4 and 6).

A possible collapse of the redox homeostasis maybe only partially involved in the observed *G*. *pps* cytotoxicity. Pre-exposure to NAC did not completely reverse the inhibition of cell proliferation and annexin V positivity (Fig 5D and 5E). Active metabolites in the extract may directly affect the mitochondria, nucleus and cell membrane. *Ganoderma tsugae* induces ROS-independent mitochondria-mediated apoptosis in human leukemia cells [46]. The cytotoxicity of steroid 9,11-dehydroergosterol peroxide from *Ganoderma lucidum* mycelium involves direct effects on mitochondria and nucleus [47]. The lipid components of *G*. *pps* may also affect the integrity of cell membrane leading to cell death. *G*. *pps* extracts may contain metal ion chelating activity as has been reported on some edible mushrooms [48]. The activation of other signal transduction pathways is another possibility and they remain to be identified. Regardless of the involvement of other mechanisms, ours is the first report on the cytotoxicity of *G*. *pps* extracts in both hematologic malignant cell lines and patient-derived cell samples, and we propose a model for the observed antineoplastic activity (Fig 9).

In summary, the activation of the ASK1-MEK-SAPK/JNK pathway is a major mechanism of the anticancer activity of the *G*. *pps* extract. What specific factors trigger this activation remain to be defined. Identification of biologically active metabolites in the *G*. *pps* extract that perturb redox homeostasis, mitochondria integrity, and nuclear functions will not only dissect other mechanisms but may provide therapeutic values for patients with hematologic malignancies.

## Supporting information

S1 Table List of primary antibodies, their sources and dilutions. (DOCX)

S1 Raw images (PDF)

## Abbreviations

AML: acute myeloid leukemia
Ann V: annexin V
CCK-8: cell counting kit-8
CM-H2DCFDA: 5-(and-6)-chloromethyl-2′,7′-dichlorodihydrofluorescein diacetate, acetyl ester
DMSO: dimethyl sulfoxide
*G*. *pps*: *Gymnopilus purpureosquamulosus*
IC_50_: inhibitory (50%) concentration
ITS: internal transcribed spacer
JC-1: 5,5′,6,6′-tetrachloro-1,1′,3,3′-tetraethylbenzimidazolyl-carbocyanine iodide
MMP: mitochondrial membrane potential
MTT: 3-(4,5 dimethylthiazol-2-yl)-2,5-diphenyl tetrazolium bromide
NAC: N-acetylcysteine
PBS: phosphate buffer saline
PCR: polymerase chain reaction
ppm: parts per million
ROS: reactive oxygen species
Z-VAD-FMK: carbobenzoxy-valyl-alanyl-aspartyl-[O-methyl]- fluoromethylketone

## References

[1] World Health Organization. WHO report on cancer: setting priorities, investing wisely and providing care for all. Geneva: World Health Organization. Retrieved from https://apps.who.int/iris/handle/10665/330745. 2020.

[2] American Cancer Society. Cancer Facts & Figures 2020. Available online at https://www.cancer.org/research/cancer-facts-statistics.html. 2020.

[3] Chaitanya MVNL, Jose A, Ramalingam P, Mandal SC, Narendra Kumar P. Multi-targeting cytotoxic drug leads from mushrooms. Asian Pac J Trop Med. 2019; 12:531–6.

[4] Sak K. Chemotherapy and dietary phytochemical agents. Chem Res Prac. 2012; 2012:282570. 10.1155/2012/282570 23320169 PMC3539428

[5] Bharali DJ, Khalil M, Gurbuz M, Simone TM, Mousa SA. Nanoparticles and cancer therapy: A concise review with emphasis on dendrimers. Int J Nanomedicine. 2009; 4:1–7. 19421366 PMC2720735

[6] Florea AM, Büsselberg D. Cisplatin as an anti-tumor drug: cellular mechanisms of activity, drug resistance and induced side effects. Cancers. 2011; 3:1351–71. 10.3390/cancers3011351 24212665 PMC3756417

[7] Xu R, Wang Q. Large-scale automatic extraction of side effects associated with targeted anticancer drugs from full-text oncological articles. J Biomed Inform. 2015; 55:64–72. 10.1016/j.jbi.2015.03.009 25817969 PMC4582661

[8] Wasser SP. Medicinal mushroom science: history, current status, future trends, and unsolved problems. Int J Med Mushrooms. 2010; 12:1–16. 10.1615/intjmedmushr.v13.i5.10 22324407

[9] Patel S, Goyal A. Recent developments in mushrooms as anticancer therapeutics: a review. 3 Biotech. 2012; 2:1–15. 10.1007/s13205-011-0036-2 22582152 PMC3339609

[10] Boukes GJ, Koekemoer TC, van de Venter M, Govender S. Cytotoxicity of thirteen South African macrofungal species against five cancer cell lines. S Afri J Bot. 2017; 113:62–7.

[11] Badalyan SM. Potential of mushroom bioactive molecules to develop healthcare biotech products. In proceeding of the 8th International Conference on Mushroom Biology and Mushroom Products. New Delhi, India. 2014.

[12] Ragasa CY, Oyong GG, Tan MCS, De Los Reyes MM, De Castro MEG. Cytotoxic sterols from Philippine mushrooms. Asian J Chem. 2020; 32:1197–202.

[13] Arnante MES, Clerigo MM, Paano AMC, Enriquez MLD. Cytotoxic and genotoxic activity of an extract from the mushroom Lenzites betulina against K562 leukemia cells. In proceeding of the DLSU Research Congress 2017, De La Salle University, Manila, Philippines. 2017.

[14] Buniel PAS, Scheewe HWP, Sanico CG Jr, Alima ZD, Demayo CG. Assessing the genotoxic and cytotoxic responses of the H-29 cancer cell lines on the ethanolic extracts of the oyster mushroom, *Pleurotus ostreatus* var. Florida. Int J Pharm Sci Res. 2018; 9:4201–9.

[15] De Castro MEG, Dulay RMR. Macrofungi in multistorey agroforestry systems in Mt. Makiling Forest Reserve, Los Banos, Laguna, Philippines. J Chem Biol Phys Sci. 2015; 5:1646–55.

[16] De Leon AM, Kalaw SP, Dulay RMD, Undan JR, Alfonzo DO, Undan JQ, et al. Ethnomycological survey of the Kalanguya indigenous community in Caranglan, Nueva Ecija, Philippines. Curr Res Environ Appl Mycol. 2016; 6:61–6.

[17] Dulay RMR, Maglasang CC. Species listing of naturally occurring mushrooms in agroecosystem of Barangay Bambanaba, Cuyapo, Nueva Ecija, Philippines. Int J Biol Pharm Allied Sci. 2017; 6:1459–72.

[18] Liwanag JMG, Santos EE, Flores FR, Clemente RF, Dulay RMR. Species listing of macrofungi in Angat Watershed Reservation, Bulacan Province, Luzon Island, Philippines. Int J Biol Pharm Allied Sci. 2017; 6:1060–8.

[19] Culala JM, Dulay RMR. Species listing of naturally occurring mushrooms in Central Luzon State University, Science City of Munoz, Nueva Ecija, Philippines. Int J Biol Pharm Allied Sci. 2018; 7:1890–9.

[20] Dulay RMR, Cabrera EC, Kalaw SP, Reyes RG. Nucleotide sequencing and identification of wild mushrooms from the Philippines. Biocatal Agric Biotechnol. 2020; 27:101666.

[21] White TJ, Bruns T, Lee S, Taylor JW. Amplification and direct sequencing of fungal ribosomal RNA genes for phylogenetics. PCR Protocols: A Guide to Methods and Applications. Edited by: Innis M. A.; Gelfand D. H.; Sninsky; White T. J. 1990, New York: Academic Press Inc, 315–322.

[22] Altschul SF, Gish W, Miller W, Myers EW, Lipman DJ. Basic local alignment search tool. J Mol Biol. 1990; 215:403–10. 10.1016/S0022-2836(05)80360-2 2231712

[23] Valdez BC, Murray D, Ramdas L, de Lima M, Jones R, Kornblau S, et al. Altered gene expression in busulfan-resistant human myeloid leukemia. Leuk Res. 2008; 32:1684–97. 10.1016/j.leukres.2008.01.016 18339423 PMC2633244

[24] Wyllie AH. Glucocorticoid induced thymocyte apoptosis is associated with endogenous endonuclease activation. Nature. 1980; 284:555–6. 10.1038/284555a0 6245367

[25] Aoki H, Kang PM, Hampe J, Yoshimura K, Noma T, Matsuzaki M, et al. Direct activation of mitochondrial apoptosis machinery by c-Jun N-terminal kinase inadult cardiac myocytes. J Biol Chem. 2002; 277:10244–50. 10.1074/jbc.M112355200 11786558

[26] Song JJ, Rhee JG, Suntharalingam M, Walsh SA, Spitz DR, Lee YJ. Role of glutaredoxin in metabolic oxidative stress. Glutaredoxin as a sensor of oxidative stress mediated by H2O2. J Biol Chem. 2002; 277:46566–75. 10.1074/jbc.M206826200 12244106

[27] Song JJ, Lee YJ. Dissociation of Akt1 from its negative regulator JIP1 is mediated through the ASK1-MEK-JNK signal transduction pathway during metabolic oxidative stress: a negative feedback loop. J Cell Biol. 2005; 170:61–72. 10.1083/jcb.200502070 15998799 PMC2171369

[28] Al-Kandari N, Fadel F, Al-Saleh F, Khashab F, Al-Maghrebi M. The thioredoxin system is regulated by the ASK-1/JNK/p38/survivin pathway during germ cell apoptosis. Molecules. 2019; 24:3333. 10.3390/molecules24183333 31547465 PMC6767173

[29] Psenakova K, Hexnerova R, Srb P, Obsilova V, Veverka V, Obsil T. The redox-active site of thioredoxin is directly involved in apoptosis signal-regulating kinase 1 binding that is modulated by oxidative stress. FEBS J. 2020; 287:1626–44. 10.1111/febs.15101 31623019

[30] Matsuzawa A, Ichijo H. Molecular mechanisms of the decision between life and death: regulation of apoptosis by apoptosis signal-regulating kinase 1. J Biochem. 2001; 130:1–8. 10.1093/oxfordjournals.jbchem.a002947 11432772

[31] Manikanta K, Naveen Kumar SK, Hemshekhar M, Kemparaju K, Girish KS. ASK1 inhibition triggers platelet apoptosis via p38-MAPK-mediated mitochondrial dysfunction. Haematologica. 2020; 105:e419–e423. 10.3324/haematol.2019.233908 31780630 PMC7395269

[32] Kim HS, Lee M-S. Essential role of STAT1 in caspase-independent cell death of activated macrophages through the p38 mitogen-activated protein kinase/STAT1/reactive oxygen species pathway. Mol Cell Biol. 2005; 25:6821–33. 10.1128/MCB.25.15.6821-6833.2005 16024814 PMC1190352

[33] Saha P, Guha S, Biswas S. C. P38K and JNK pathways are induced by amyloid-β in astrocyte: Implication of MAPK pathways in astrogliosis in Alzheimer’s disease. Mol Cell Neurosci. 2020; 108:103551. 10.1016/j.mcn.2020.103551 32896578

[34] Durgo K, Koncar M, Komes D, Belscak-Cvitanovic A, Franekic J, Jakopovich I, et al. Cytotoxicity of blended versus single medicinal mushroom extracts on human cancer cell lines: contribution of polyphenol and polysaccharide content. Int J Med Mushrooms. 2013; 15:435–8. 10.1615/intjmedmushr.v15.i5.20 24266369

[35] Joseph TP, Chanda W, Padhiar AA, Batool S, LiQun S, Zhong M, et al. A preclinical evaluation of the antitumor activities of edible and medicinal mushrooms: A molecular insight. Integr Cancer Ther. 2018; 17:200–9. 10.1177/1534735417736861 29094602 PMC6041903

[36] Zhang M, Zhang Y, Zhang L, Tian Q. Mushroom polysaccharide lentinan for treating different types of cancers: A review of 12 years clinical studies in China. Prog Mol Biol Transl Sci. 2019; 163:297–328. 10.1016/bs.pmbts.2019.02.013 31030752

[37] Liu Y, Min W. Thioredoxin promotes ASK1 ubiquitination and degradation to inhibit ASK1-mediated apoptosis in a redox activity-independent manner. Circ Res. 2002; 90:1259–66. 10.1161/01.res.0000022160.64355.62 12089063

[38] Zhang J, Jing X, Niu W, Zhang M, Ge L, Miao C, et al. Peroxiredoxin 1 has an anti-apoptotic role via apoptosis signal-regulating kinase 1 and p38 activation in mouse models with oral precancerous lesions. Oncol Lett. 2016; 12:413–20. 10.3892/ol.2016.4659 27347160 PMC4907023

[39] Nadeau PJ, Charette S, Toledano MB, Landry J. Disulfide bond-mediated multimerization of Ask1 and its reduction by thioredoxin-1 regulate H(2)O(2)-induced c-Jun NH(2)-terminal kinase activation and apoptosis. Mol Biol Cell. 2007; 18:3903–13. 10.1091/mbc.e07-05-0491 17652454 PMC1995733

[40] Soethoudt M, Peskin AV, Dickerhof N, Paton LN, Pace PE, Winterbourn CC. Interaction of adenanthin with glutathione and thiol enzymes: selectivity for thioredoxin reductase and inhibition of peroxiredoxin recycling. Free Radic Biol Med. 2014; 77:331–9. 10.1016/j.freeradbiomed.2014.09.025 25289458

[41] Matsuzawa A, Ichijo H. Redox control of cell fate by MAP kinase: physiological roles of ASK-MAP kinase pathway in stress signaling. Biochim Biophys Acta. 2008; 1780:1325–36. 10.1016/j.bbagen.2007.12.011 18206122

[42] Dhanasekaran DN, Reddy EP. JNK-signaling: A multiplexing hub in programmed cell death. Genes Cancer. 2017; 8:682–94. 10.18632/genesandcancer.155 29234486 PMC5724802

[43] Papadakis ES, Finegan KG, Wang X, Robinson AC, Guo C, Kayahara M, et al. The regulation of Bax by c-Jun N-terminal protein kinase (JNK) is a prerequisite to the mitochondrial-induced apoptotic pathway. FEBS Lett. 2006; 580:1320–6. 10.1016/j.febslet.2006.01.053 16458303

[44] Fischer U, Janicke RU, Schulze-Osthoff K. Many cuts to ruin: a comprehensive update of caspase substrates. Cell Death Differ. 2003; 10:76–100. 10.1038/sj.cdd.4401160 12655297 PMC7091709

[45] Fang EF, Scheibye-Knudsen M, Chua KF, Mattson MP, Croteau DL, Bohr VA. Nuclear DNAdamage signaling to mitochondria in ageing. Nat Rev Mol Cell Biol. 2016; 17:308–21. 10.1038/nrm.2016.14 26956196 PMC5161407

[46] Hseu Y-C, Shen Y-C, Kao M-C, Mathew DC, Karuppaiya P, Li M-L, et al. *Ganoderma tsugae* induced ROS-independent apoptosis and cytoprotective autophagy in human chronic myeloid leukemia cells. Food Chem Toxic. 2019; 124:30–44. 10.1016/j.fct.2018.11.043 30465897

[47] Zheng L, Wong Y-S, Shao M, Huang S, Wang F, Chen J. Apoptosis induced by 9,11-dehydroergosterol peroxide from *Ganoderma lucidum* mycelium in human malignant melanoma cells in Mcl-1 dependent. Mol Med Rep. 2018; 18:938–44. 10.3892/mmr.2018.9035 29845223 PMC6059726

[48] Sadi G, Emsen B, Kaya A, Kocabas A, Cinar S, Kartal DI. Cytotoxicity of some edible mushrooms extracts over liver hepatocellular carcinoma cells in conjunction with their antioxidant and antibacterial properties. Pharma Mag. 2015; 11:S6–S18. 10.4103/0973-1296.157665 26109775 PMC4461969

