# Supplementary material for: Cytotoxicity of Gymnopilus purpureosquamulosus extracts on hematologic malignant cells through activation of the SAPK/JNK signaling pathway
Source: PLoS One. 2021 May 28;16(5):e0252541. doi: 10.1371/journal.pone.0252541 (PMC8162692; doi:10.1371/journal.pone.0252541)
Supplement: S1 Table — (DOCX) [file pone.0252541.s001.docx]

| S1 Table. List of primary antibodies, their sources and dilutions | | | | |
| --- | --- | --- | --- | --- |
| **Antigen** | **Company/Cat. #** | **Source** | **Dilution*** |  |
| AcH3K9 | Active&Motif#39137 | Rabbit | 3500 |  |
| AcH3K18 | Active&Motif#39694 | Rabbit | 3500 |  |
| β-Actin | Sigma/A5316 | Mouse | 6000 |  |
| AIF | Cell Signaling/5318 | Rabbit | 2000 |  |
| ASK1 | Cell Signaling/8662 | Rabbit | 2500 |  |
| P-ATF2 (Thr71) | Cell Signaling/5112 | Rabbit | 2500 |  |
| ATF2 | Cell Signaling/9226 | Rabbit | 2500 |  |
| BAX | Santa Cruz Biotech/526 | Rabbit | 1500 |  |
| BIM | Cell Signaling/2933 | Rabbit | 2000 |  |
| Caspase 3 | Cell Signaling/9661 | Rabbit | 2500 |  |
| Caspase 9 | Cell Signaling/9502 | Rabbit | 2500 |  |
| Cytochrome c | BD PharMingen/556433 | Mouse | 1500 |  |
| P-ERK1/2 (Thr202/Ty204) | Cell Signaling/9101 | Rabbit | 2500 |  |
| ERK1/2 | Cell Signaling/9102 | Rabbit | 2500 |  |
| γ-H2AX | Cell Signaling/9718 | Rabbit | 5000 |  |
| P-c-JUN(S73) | Cell Signaling/9164 | Rabbit | 2000 |  |
| c-JUN | Cell Signaling/9165 | Rabbit | 2500 |  |
| P-MAPKAPK2 (Thr334) | Cell Signaling/3041 | Rabbit | 2000 |  |
| MAPKAPK2 | Cell Signaling/3042 | Rabbit | 2500 |  |
| MCL-1 | Santa Cruz/819 | Rabbit | 1000 |  |
| 3MeH3K27 | Active&Motif#39155 | Rabbit | 3500 |  |
| P-MEK1/2 (Ser217/Ser221) | Cell Signaling/9154 | Rabbit | 2000 |  |
| MEK1/2 | Cell Signaling/8727 | Rabbit | 2500 |  |
| c-MYC | Cell Signaling/9402 | Rabbit | 3500 |  |
| PARP1 | Santa Cruz Biotech/8007 | Mouse | 1000 |  |
| cleaved PARP1 | Cell Signaling/5625 | Rabbit | 3500 |  |
| Peroxiredoxin 1 | upstate/07-609 | Rabbit | 2500 |  |
| P-P38 (Thr180/Tyr182) | Cell Signaling/2915 | Rabbit | 2500 |  |
| P-38 | Cell Signaling/9212 | Rabbit | 2500 |  |
| P-SAPK/JNK (Thr183/Tyr185) | Cell Signaling/4668 | Rabbit | 2000 |  |
| SAPK/JNK | Cell Signaling/9258 | Rabbit | 2500 |  |
| P-STAT1 (Tyr701) | Cell Signaling/7649 | Rabbit | 2000 |  |
| STAT1 | Cell Signaling/9172 | Rabbit | 3500 |  |
| Thioredoxin | Cell Signaling/2429 | Rabbit | 2000 |  |
| Thioredoxin reductase | Cell Signaling/6925 | Rabbit | 2000 |  |
| XAF1 | Santa Cruz/19193 | Goat | 700 |  |
| XIAP | Cell Signaling/2045 | Rabbit | 3000 |  |
| *Used anti-rabbit IgG for secondary antibody from Bio-Rad Lab. | | | |  |
| Used anti-mouse IgG for secondary antibody from Bio-Rad Lab. | | | |  |
| **Fold dilution in PBS with 0.05% Tween 20 | |  |  |  |
