# Supplementary material for: Cytotoxicity of Gymnopilus purpureosquamulosus extracts on hematologic malignant cells through activation of the SAPK/JNK signaling pathway
Source: PLoS One. 2021 May 28;16(5):e0252541. doi: 10.1371/journal.pone.0252541 (PMC8162692; doi:10.1371/journal.pone.0252541)
Supplement: S1 Raw images — (PDF) [file pone.0252541.s002.pdf]

**Figure 4A Raw images**

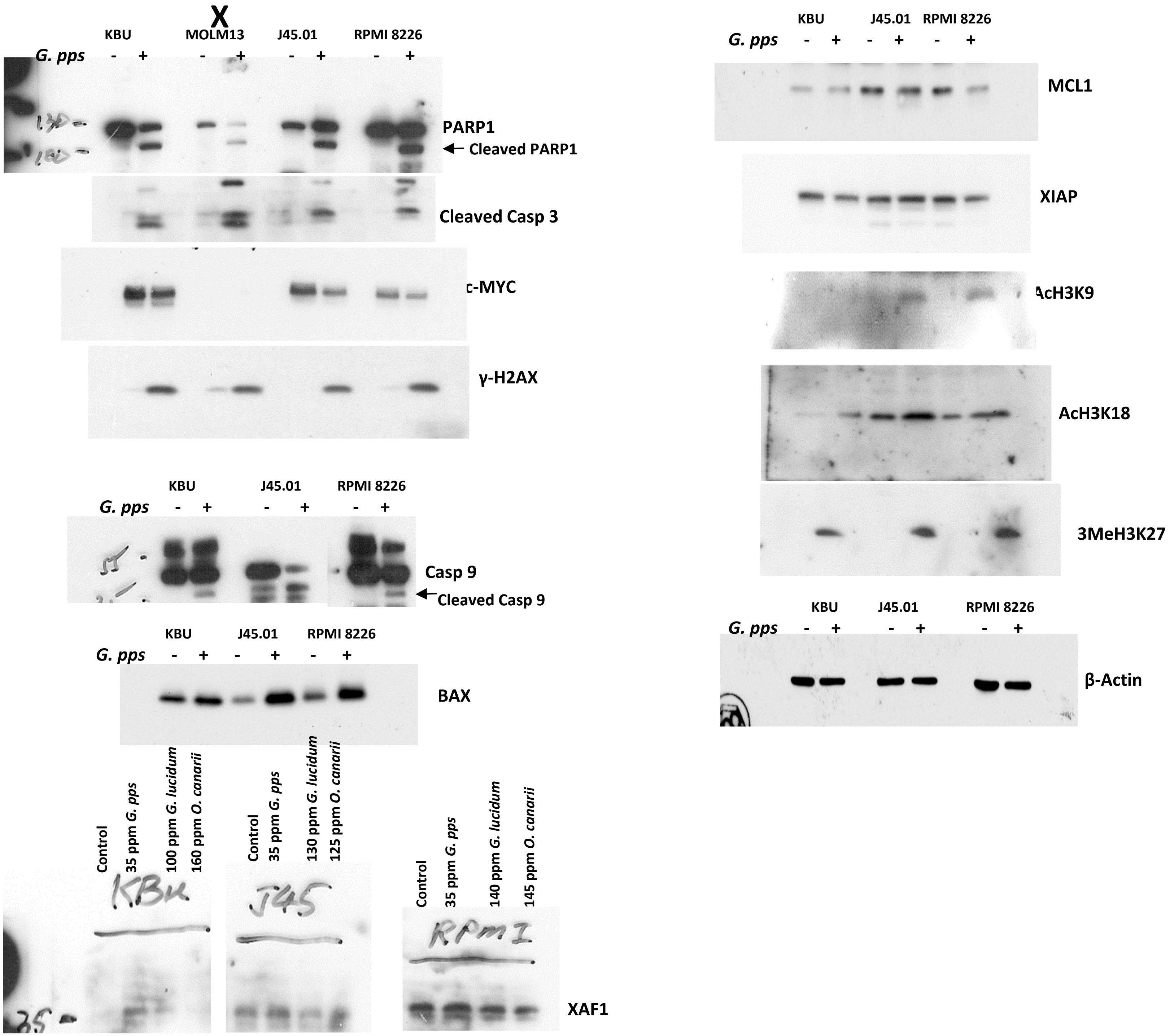

**Figure 4B. Isolated genomic DNA on agarose gel**

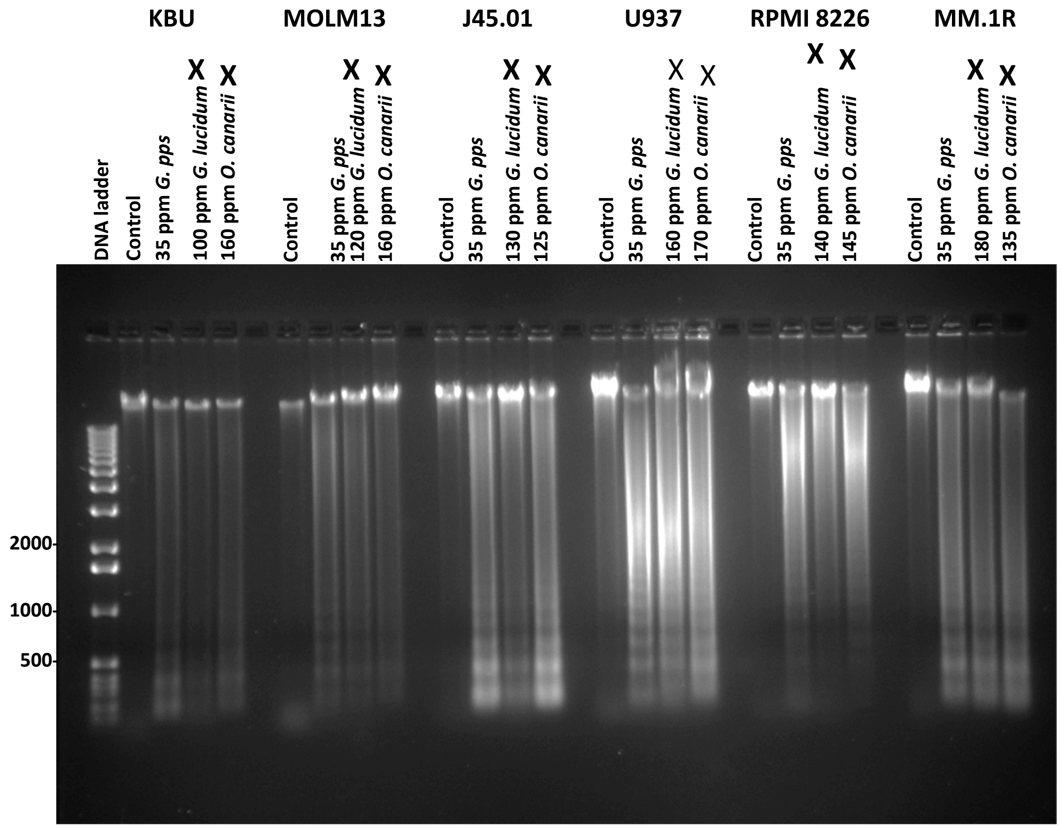

**Figure 4C**

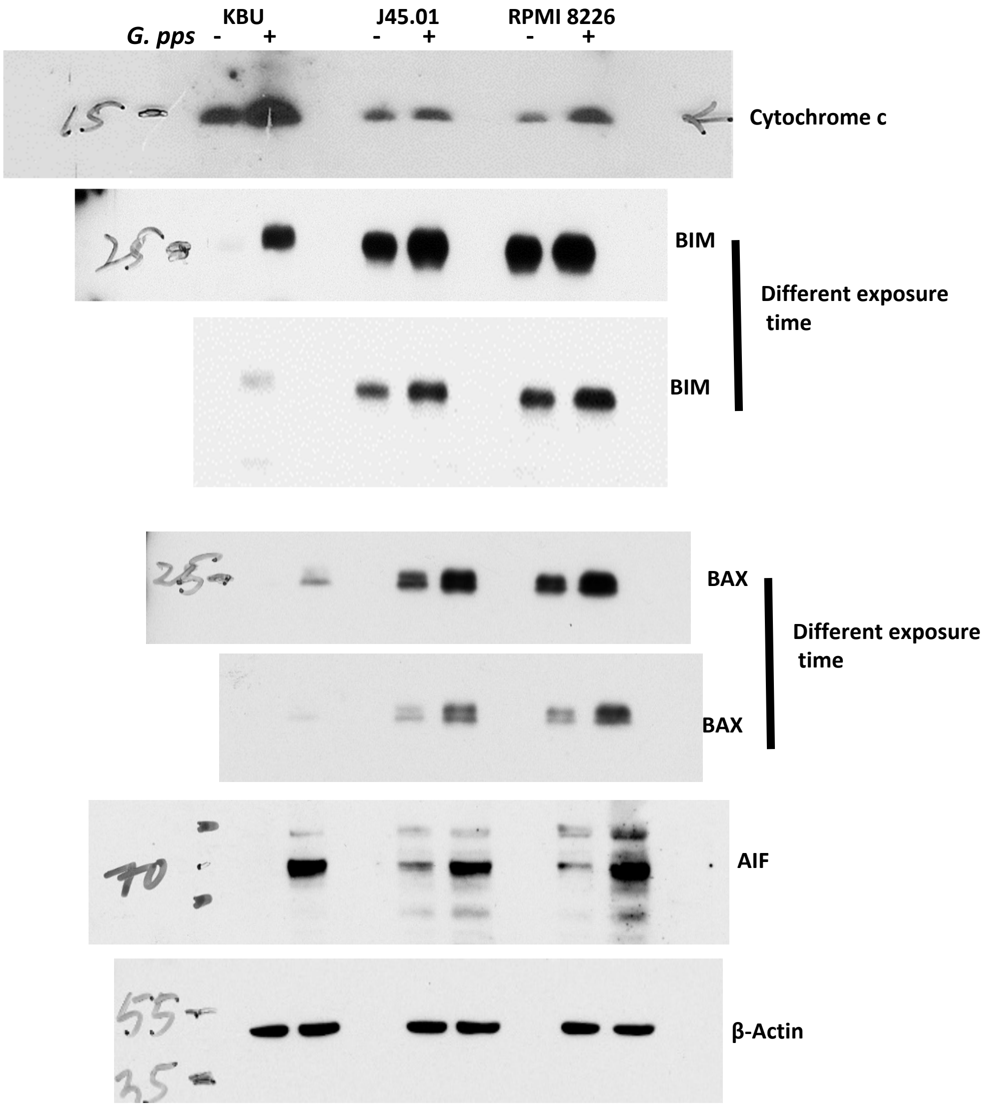

KBU
J45.01
RPMI 8226
G. pps
-
+
-
+
-
+
15
Cytochrome c
25
BIM
Different exposure time
BIM
25
BAX
Different exposure time
BAX
70
AIF
55
35
β-Actin

**Figure 5C**

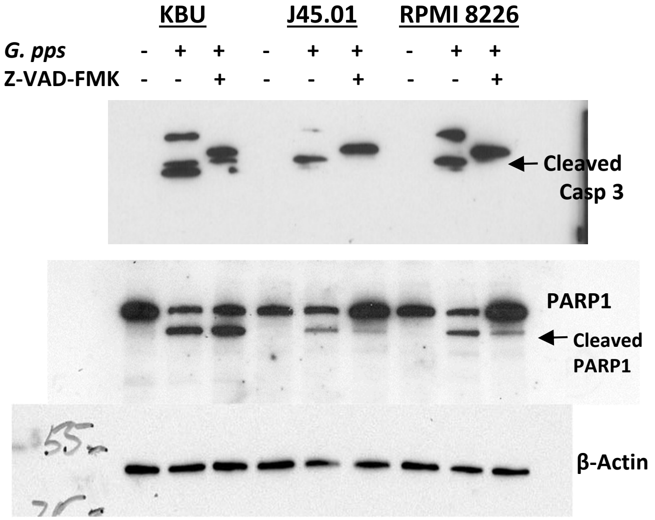


**Figure 5F**

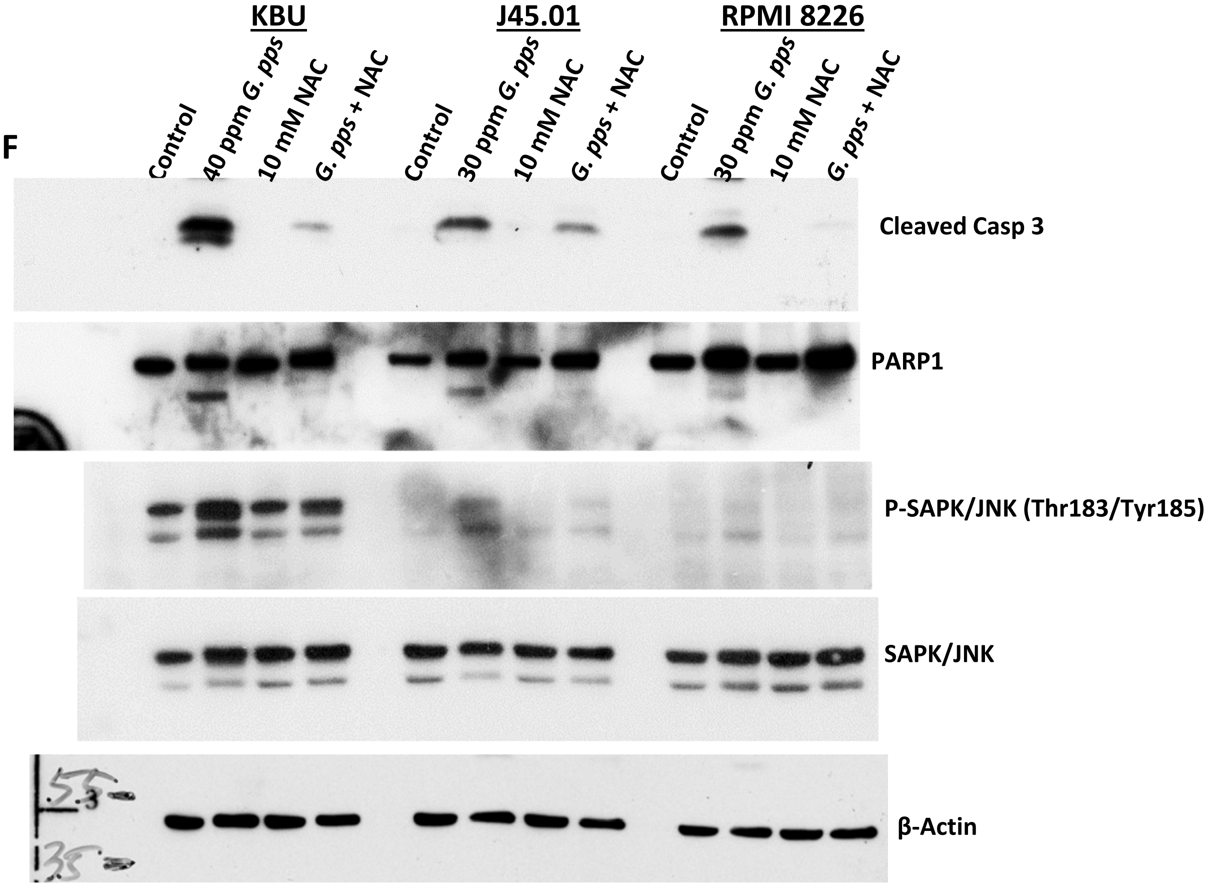

Figure 7A

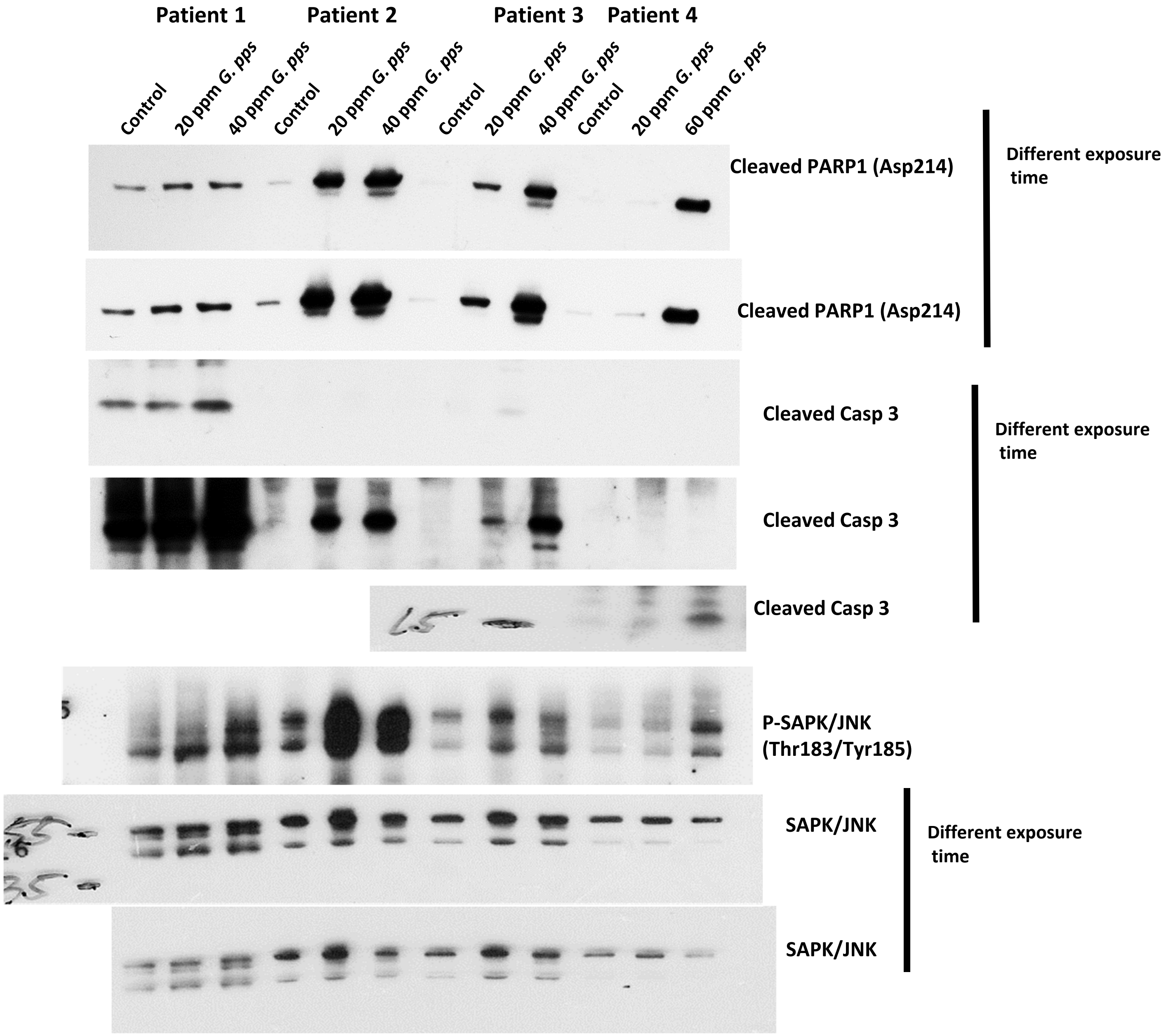
Patient 1
Patient 2
Patient 3
Patient 4
Control
20 ppm G. pps
40 ppm G. pps
Control
20 ppm G. pps
40 ppm G. pps
Control
20 ppm G. pps
40 ppm G. pps
Control
20 ppm G. pps
60 ppm G. pps
Cleaved PARP1 (Asp214)
Cleaved PARP1 (Asp214)
Different exposure time
Cleaved Casp 3
Cleaved Casp 3
Cleaved Casp 3
Different exposure time
P-SAPK/JNK (Thr183/Tyr185)
SAPK/JNK
SAPK/JNK
Different exposure time

**Figure 7A** cont'd

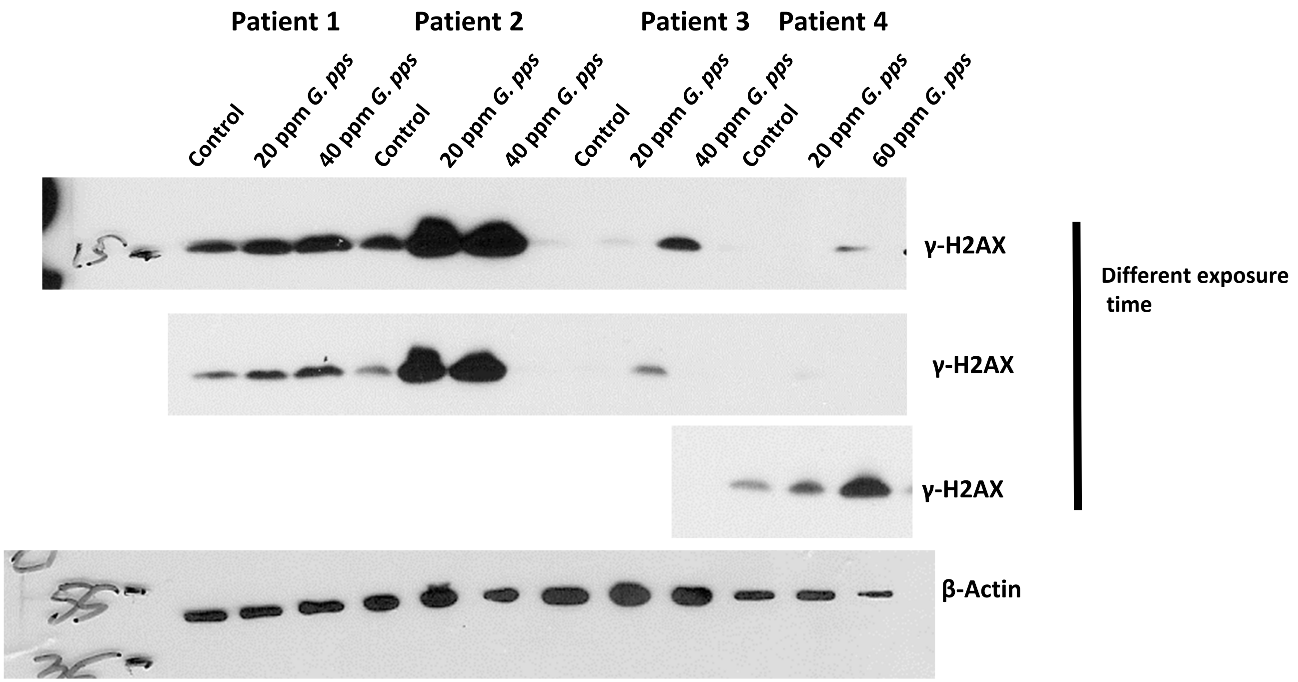


**Figure 7B**

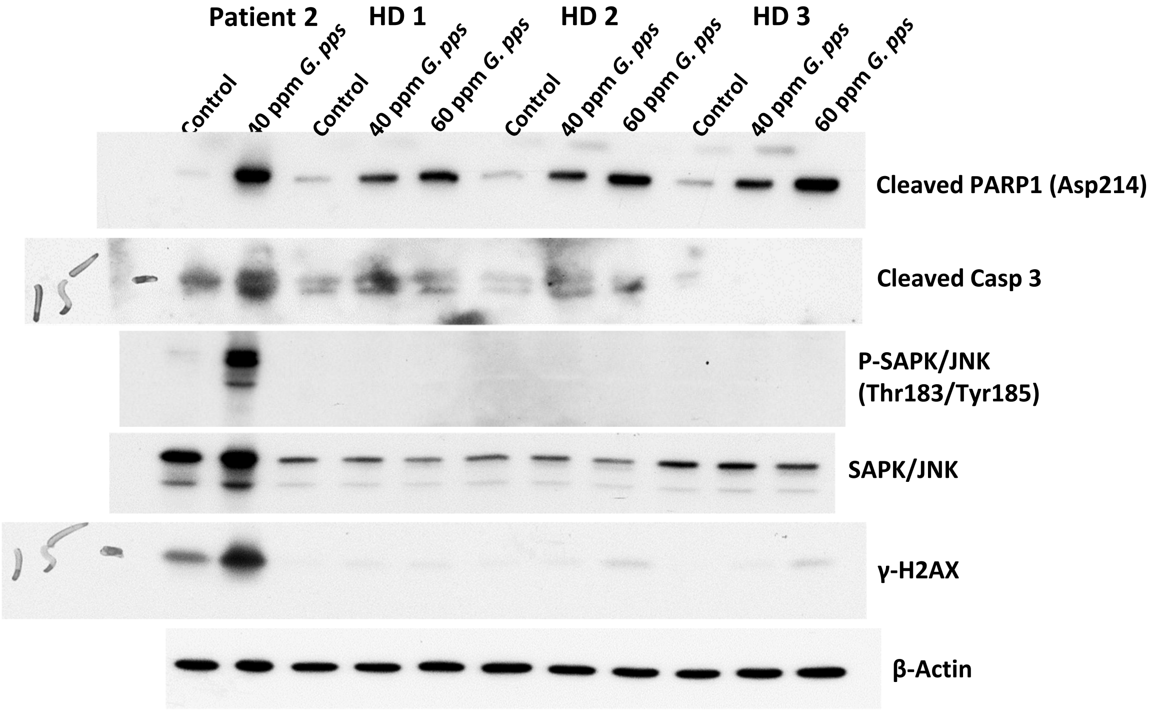

**Figure 8A**

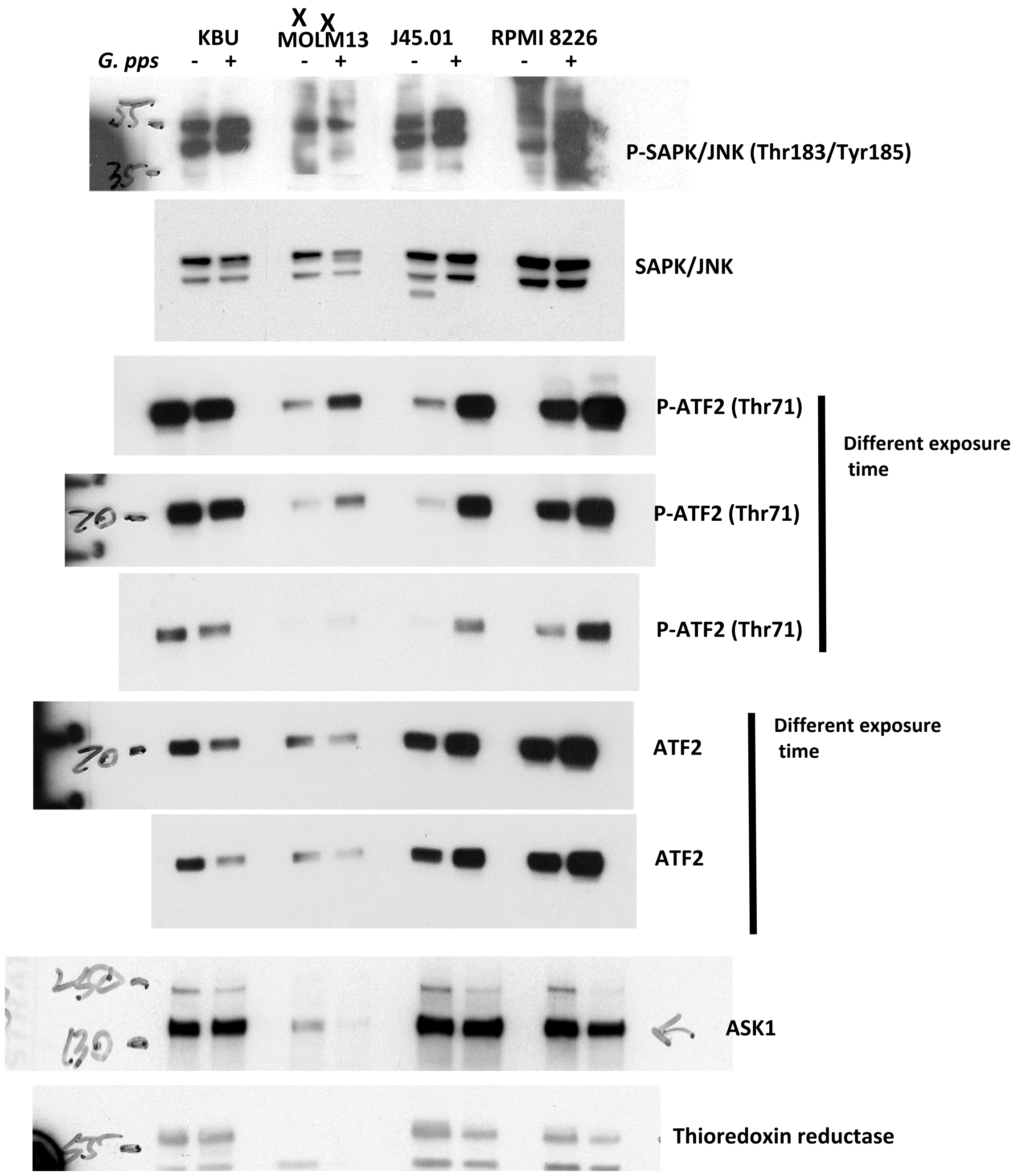
KBU
MOLM13
J45.01
RPMI 8226
G. pps
- + - + - + - +
55
35
P-SAPK/JNK (Thr183/Tyr185)
SAPK/JNK
P-ATF2 (Thr71)
P-ATF2 (Thr71)
P-ATF2 (Thr71)
Different exposure time
70
ATF2
ATF2
Different exposure time
250
130
ASK1
55
Thioredoxin reductase

**Figure 8A continued**

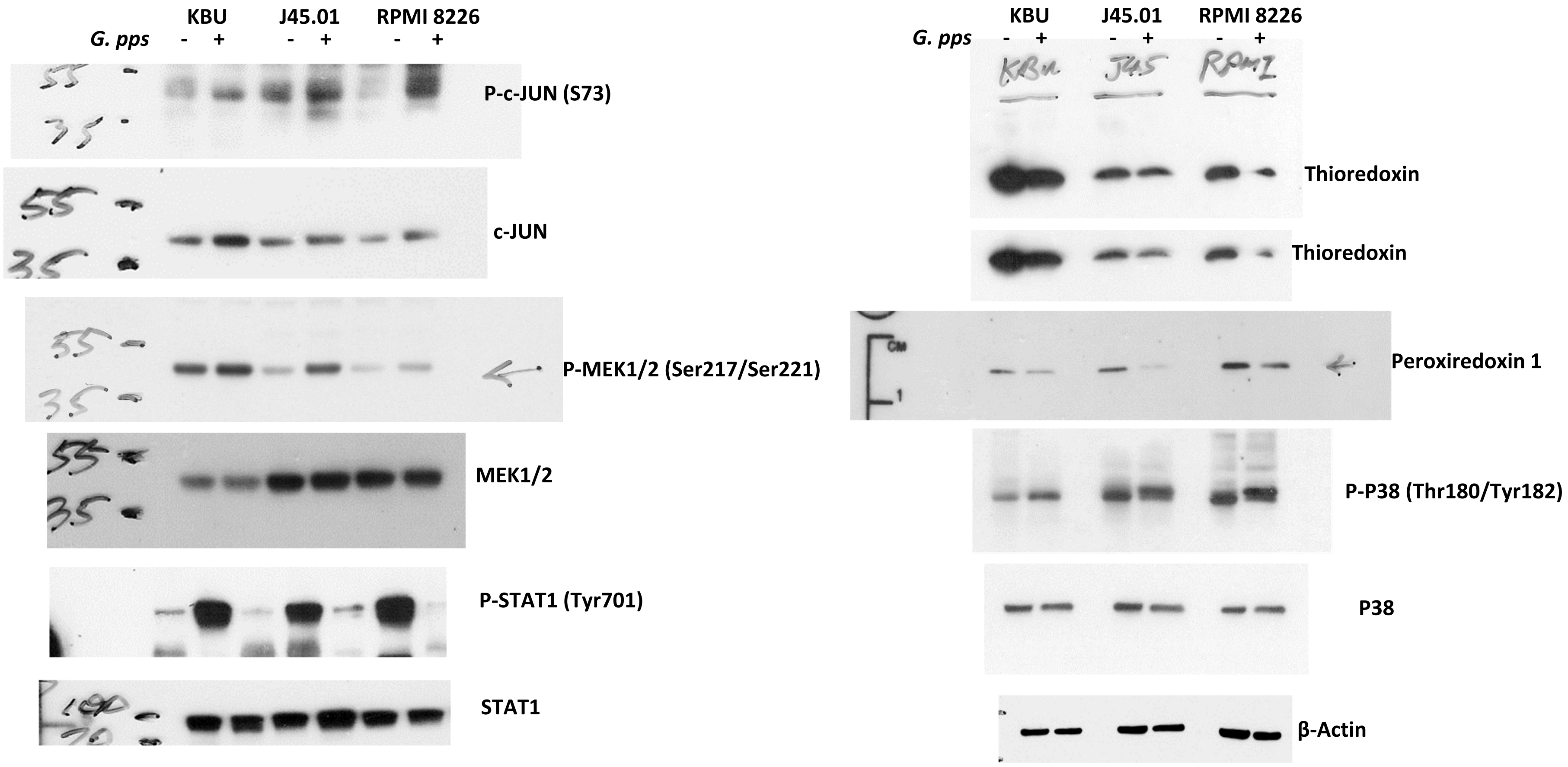

**Figure 8B**

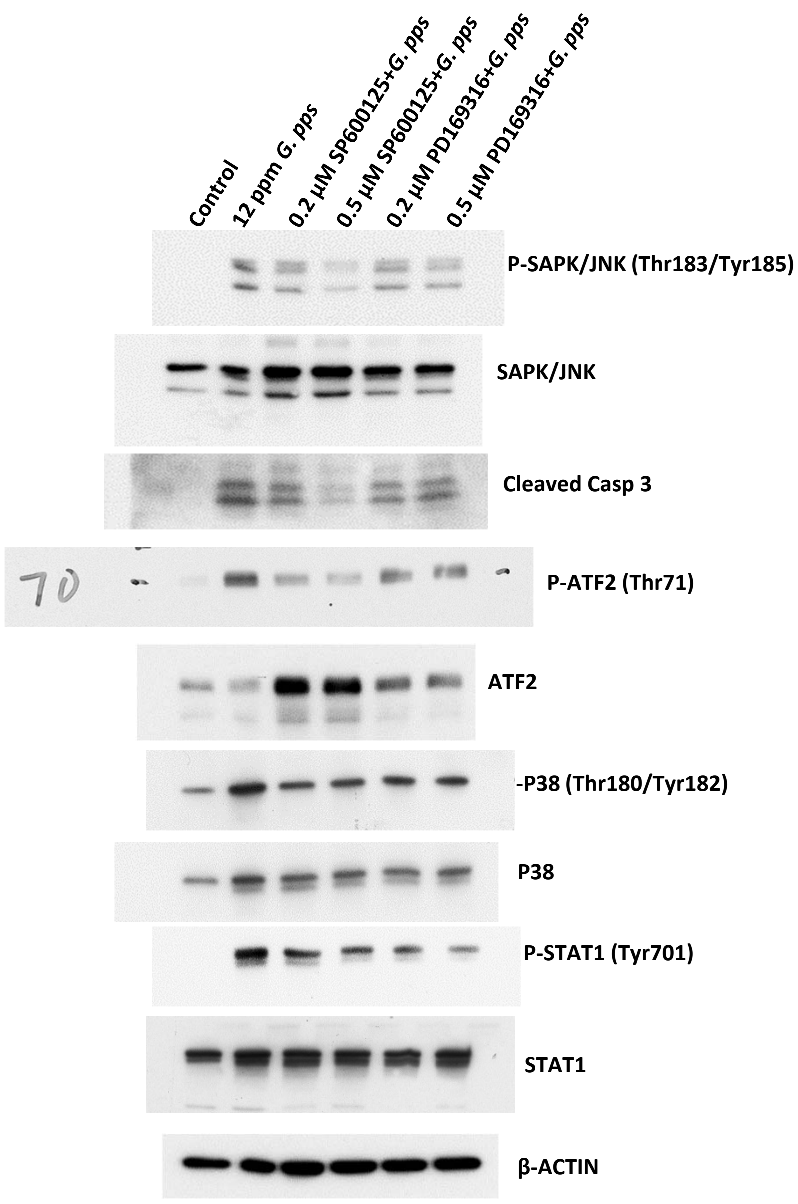
Control
12 ppm G. pps
0.2 µM SP600125+G. pps
0.5 µM SP600125+G. pps
0.2 µM PD169316+G. pps
0.5 µM PD169316+G. pps
P-SAPK/JNK (Thr183/Tyr185)
SAPK/JNK
Cleaved Casp 3
70
P-ATF2 (Thr71)
ATF2
-P38 (Thr180/Tyr182)
P38
P-STAT1 (Tyr701)
STAT1
β-ACTIN
